# Microbes control *Drosophila* germline stem cell increase and egg maturation through hormonal pathways

**DOI:** 10.1038/s42003-023-05660-x

**Published:** 2023-12-20

**Authors:** Ritsuko Suyama, Nicolas Cetraro, Joanne Y. Yew, Toshie Kai

**Affiliations:** 1 Laboratory of Germline Biology, Graduate School of Frontier Biosciences, Osaka University, 1-3 Yamadaoka Suita, Osaka, 565-0871 Japan; 2 Pacific Biosciences Research Center, University of Hawai’i at Manoa, 1993 East-West Road, Honolulu, HI 96822 USA

**Keywords:** Oogenesis, Microbiome, Stem-cell differentiation

## Abstract

Reproduction is highly dependent on environmental and physiological factors including nutrition, mating stimuli and microbes. Among these factors, microbes facilitate vital functions for host animals such as nutritional intake, metabolic regulation, and enhancing fertility under poor nutrition conditions. However, detailed molecular mechanisms by which microbes control germline maturation, leading to reproduction, remain largely unknown. In this study, we show that environmental microbes exert a beneficial effect on *Drosophila* oogenesis by promoting germline stem cell (GSC) proliferation and subsequent egg maturation via acceleration of ovarian cell division and suppression of apoptosis. Moreover, insulin-related signaling is not required; rather, the ecdysone pathway is necessary for microbe-induced increase of GSCs and promotion of egg maturation, while juvenile hormone contributes only to increasing GSC numbers, suggesting that hormonal pathways are activated at different stages of oogenesis. Our findings reveal that environmental microbes can enhance host reproductivity by modulating host hormone release and promoting oogenesis.

## Abstract (web summary)

Environmental microbes enhance *Drosophila* oogenesis by increasing the number of germline stem cells, suppressing apoptosis and accelerating ovarian cell division through hormonal pathways.

## Introduction

Environmental factors, such as stress, age, nutritional status, and mating, can have a large effect on animal physiology by regulating homeostatic systems^1,2^. Among environmental influences, the microbiome, the community of fungi and bacteria in host organisms, exerts enormous influence on vital functions such as nutrient intake, immune responses, metabolic homeostasis and reproduction^3–9^. In particular, reproduction, which requires more energy than any other physiological function, is tightly regulated by both environmental signals and internal homeostatic mechanisms.

*Drosophila* oogenesis is initiated by germline stem cells (GSCs) localized at the anterior of the germarium. Maintaining stem cell lineage depends on local signals from the proximal somatic microenvironment, a niche composed of cap cells, escort stem cells and terminal filament cells^10^. GSCs proliferate and differentiate into 16-cell cysts, resulting in an oocyte and 15 nurse cells after four rounds of incomplete cytokinesis in the germarium^10^. Germ cysts are then enveloped by the somatic follicle cells and the oocyte becomes a mature egg by receiving essential substances, such as organelles, mRNAs, and proteins from nurse cells^11,12^. During egg maturation, programmed cell death (PCD) acts as a checkpoint at the germarium and mid-stage, modulating the entire oogenesis process, including egg production^13,14^. These processes are controlled by several environmental factors via three major hormonal systems: insulin-like peptides, 20-hydroxyecdysone (20E), and juvenile hormone (JH). Receptors for these hormones are expressed in the ovarian nurse and follicle cells of the ovary^15–18^, and a lack of these hormones causes deterioration of oogenesis^1^.

Germline development and fecundity are influenced by nutritional status and mating. Their regulatory molecular mechanisms of ovarian development and inherent hormonal pathways are well characterized^1,9^. Female flies reared with a nutrition-rich diet lay more eggs and proliferate more GSCs than those in poor nutrition conditions^2,14,19–23^. Particularly, rich nutrition promotes oogenesis via *Drosophila* insulin-like peptides (*dilps*) that are produced in brain neuroendocrine cells and the gut^19,20^. In contrast, mating enhances oogenesis by activating neural pathways via ecdysone signaling^24,25^. The steroid hormone, 20-hydroxyecdysone (20E) is produced in the adult reproductive organs, gut, and head and helps to coordinate metabolic state with GSC and follicle cell maintenance and proliferation, and promote vitellogenesis during oogenesis^15,22,26–35^.

Microbes modulate host homeostasis and influence host physiology, resulting in trade-offs between reproduction and longevity^7^. Inoculating *Drosophila* with several different species of microbes improves fertility or prolongs longevity, indicating that individual microbe strains can control host physiology in distinct ways^3,7^. Microbes also accelerate embryonic maturation and metabolic homeostasis via alcohol dehydrogenase to enhance egg production^3,36^. However, little is known about the detailed molecular mechanisms of host reproduction and oogenesis that are influenced by microbes.

Here, we dissect the molecular and cellular mechanisms by which microbes influence oogenesis in *D. melanogaster*. Genetic analysis revealed that microbes enhance oogenesis through multiple mechanisms: GSC proliferation accompanied by activation of mitotic division, repression of cell death at two critical developmental checkpoints, and acceleration of the cell division of germline and follicle cells. Furthermore, the ecdysone hormone pathway appears to be a key mediator of microbe-induced processes during oogenesis at the GSC and later vitellogenic stages. In contrast, the juvenile hormone pathway is involved in GSC proliferation. We propose that microbes regulate different stages of oogenesis, possibly by modulating hormone levels and their subsequent pathways, and are able to contribute to host fecundity under poor nutrition conditions.

## Results

### Environmental microbes regulate oogenesis by egg maturation

**Fig. 1:**
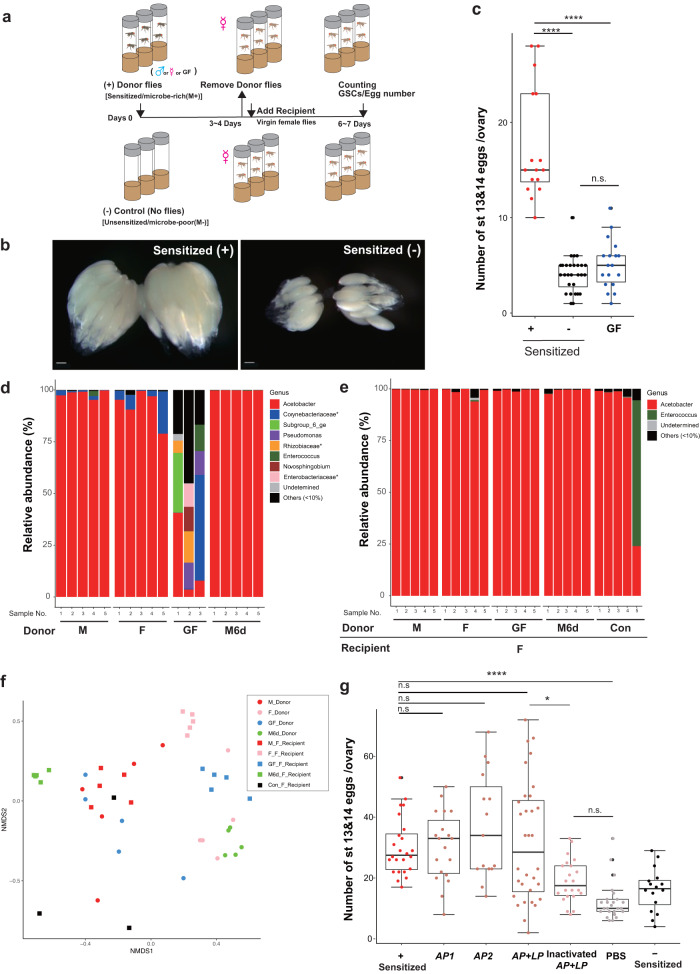
*Drosophila* oogenesis is enhanced in fly-sensitized vials. **a** Schematic drawing of the procedure for generating sensitized vials and examining female oogenesis. Vials were first sensitized by placing male pupae in fresh vials. Eclosed adult males were removed after 3-4 days. Next, female pupae were placed in the sensitized vials. After 3 days, the ovaries of eclosed adults were dissected to evaluate oogenesis. **b** Images for ovaries dissected from females cultured in sensitized vials for 3 days (Sensitized+) had more stage 13/14 eggs compared with those from females cultured in unsensitized vials (Sensitized-). Scale bar = 500 μm. **c** The number of stage 13/14 eggs in the ovaries of females cultured under different conditions: sensitized with wild-type males (CS9515, Sensitized + ), no flies (Sensitized-) or germ-free flies (GF). **d** Relative abundance profiles of microbes from donor flies (male [M], female [F] or GF flies [GF]) after 3 days’ culture in the vials. Male donor flies cultured for 6 days were also examined (M6d); (*n* = 5). **e** Microbial profiles of recipient female flies after 3 days’ culture in vials sensitized with males for 3 days [M], females for 3 days [F], GF for 3 days [GF], males for 6 days [M6d] or unsensitized [Con]; (*n* = 5). **d, e** Only genera with relative abundance above 10% are shown. Unclassified species are denoted by *. **f** Non-multidimensional scaling (NMDS) plot comparing the beta diversity of microbial compositions shown in (**d**) and (**e**). **g** The number of stage 13/14 eggs per ovary from females cultured for 3 days in sensitized vials under the following conditions: wild-type male (Sensitized + ), cultured *Acetobacter pomorum (AP)*, cultured co-application of *AP* and *Lactobacillus plantarum, LP (AP* + *LP)*, heat-inactivated *AP* + *LP* (inact *AP* + *LP*), PBS, or unsensitized (Sensitized-). For statistical analysis, a Wilcoxon rank sum test is used for (**c**) and (**g**). ****P ≤ 0.001, and *P ≤ 0.05, n.s., nonsignificant (*P* > 0.05). Data are represented as mean ± standard deviation.

*Drosophila* acquires microbes from environmental sources including dietary substrates, frass, and other drosophilids^6,37^. To elucidate the role of environmentally-acquired microbes on oogenesis, we first quantified mature eggs from “recipient” virgin females placed in vials either sensitized (microbe-rich, M + , described later) with “donor” flies or left unsensitized (microbe-poor, M-, described later). Flies were initially placed in vials for 3-4 days to ‘sensitize’ the vials and replaced with females exposed to the vials for 3 days (Fig. 1a). Consistent with the larger ovary size (Fig. 1b), females cultured in sensitized vials produced more eggs at stage 13/14 than those in unsensitized vials (Fig. 1c). The number of mature eggs (stage 13/14) were maximized when five flies were used as donors in a single vial and after 3 days of exposure for “recipient” females (Supplementary Fig. 1a, b). These conditions were used for subsequent studies. Sensitized females upon mating produced more progeny than those without sensitization, but the hatching rate was comparable under both conditions, suggesting that the quality of eggs from sensitized females was unaffected (Supplementary Fig. 1c, d).

Both pheromones and microbes deposited by flies can contribute to the oogenesis-promoting effect^3,7,38^. Several lines of evidence indicate that microbes, rather than sex-specific pheromones, underlie oogenesis enhancement. First, swab-wash eluate from sensitized vials was capable of enhancing mature egg numbers, but the effect was abolished with heat treatment (Supplementary Fig. 1e). Second, sensitizing vials with either males or females increased egg production to a similar degree (Supplementary Fig. 1f), indicating that the factors for enhancing egg maturation are not sex-specific. Third, mutant females lacking pheromone-responsive or chemo-sensitive receptors (*Or83b*, *Wnt6a*, or *Voila*^*1*^*)*^39,40^ exhibit enhanced egg maturation when placed in sensitized vials (Supplementary Fig. 1g). Lastly, vials sensitized with pheromone extract from flies did not affect egg numbers (Supplementary Fig. 1h). These results suggest that heat-susceptible factors other than sex-specific pheromones from “donor” flies were responsible for enhancing egg development in “recipient” females.

To investigate whether acquired microbes enhance egg maturation, we generated microbe-free flies (germ-free, GF)^41^ and examined their ability as donors to increase oogenesis. Notably, female flies cultured in vials sensitized by GF flies had fewer stage 13/14 eggs than those in vials sensitized by wildtype laboratory-reared flies, and the number was similar to that of flies placed in non-sensitized conditions (Fig. 1c). Indeed, we observed a positive correlation between the numbers of microbes (as measured by CFUs) and mature eggs induced by the donor flies (Supplementary Fig. 1a, b, Supplementary Table 1). In addition, no microbes were detected in germ free donor flies (Supplementary Table 1). Taken together, these findings indicate that microbes or the metabolites from donor flies are responsible for enhancing egg development in recipient female flies.

### *Acetobacter*, the primary component of laboratory-reared fly microbiomes, enhances oogenesis

To identify the genus of microbes enhancing oogenesis, we performed 16S rRNA amplicon sequencing on laboratory-reared flies used in our study. The microbiome profile was slightly more diverse in flies at the initial stage, but was dominated by *Acetobacter* between 3-6 days of age (Supplementary Fig. 2a, b). Donor flies, except for GF flies, exhibited similar profiles, with *Acetobacter* being the most abundant genus in both males and females (Fig. 1d). In contrast, the microbiome of GF flies was more diverse despite the absence of live microbes, as evident from the higher alpha-diversity score (Fig.1d, Supplementary Fig. 2a). Recipient females cultured in sensitized vials had similar composition, with *Acetobacter* as the dominant genus (Fig. 1e). We quantified the microbial load of donor or recipient flies based on counts of colony forming units (CFUs) after plating. Male and female donors at 3 d old had more microbes than GF or 6-day old flies. Moreover, the recipient females that were cultured in vials sensitized with donors for 3 days yielded more CFUs than in non-sensitized vials or ones conditioned with GF or 6 day old flies (Supplementary Table 1). The beta-diversity plot shows the transition of the microbe profile between the recipient and donor flies over time and reveals that flies cultured under the same condition have similar microbial composition (Fig. 1f).

Next, to address whether microbes can directly enhance oogenesis, we inoculated the vials with *Acetobacter, Lactobacillus*, or both taxa and examined the effect on females. The number of mature eggs from females in vials containing *Acetobacter pomorum* alone was comparable to those of females cultured in male-sensitized vials, indicating that *A.pomorum* was capable of promoting oogenesis without being deposited by flies (Fig. 1g). Importantly, heat inactivation of the microbes failed to induce egg maturation (Fig. 1g), resulting in reduced egg numbers similar to those found with heat inactivated swab-wash eluate (Supplementary Fig. 1e). Thus, microbial metabolic activity is essential for enhancing oogenesis. *Lactobacillus*, another commonly found microbe in lab-cultured flies (Supplementary Fig. 2c), with food containing tertial-butyl hydroxy peroxide (t-BH), an oxidizing agent suitable for *Lactobacillus plantarum or Lactobacillus brevis* growth^42^, enhanced egg maturation (Supplementary Fig. 2d). No synergistic effects on egg maturation between *L. plantarum* and *A.pomorum* were observed (Fig. 1g). Taken together with the outcomes of the 16 S profiling, these results indicate that major components of the fly microbiome, *Acetobacter* and *Lactobacillus*, were deposited by donor flies and were the major factors contributing to egg maturation in females placed in fly-sensitized vials.

### Microbes enhance egg maturation and GSC numbers

**Fig. 2:**
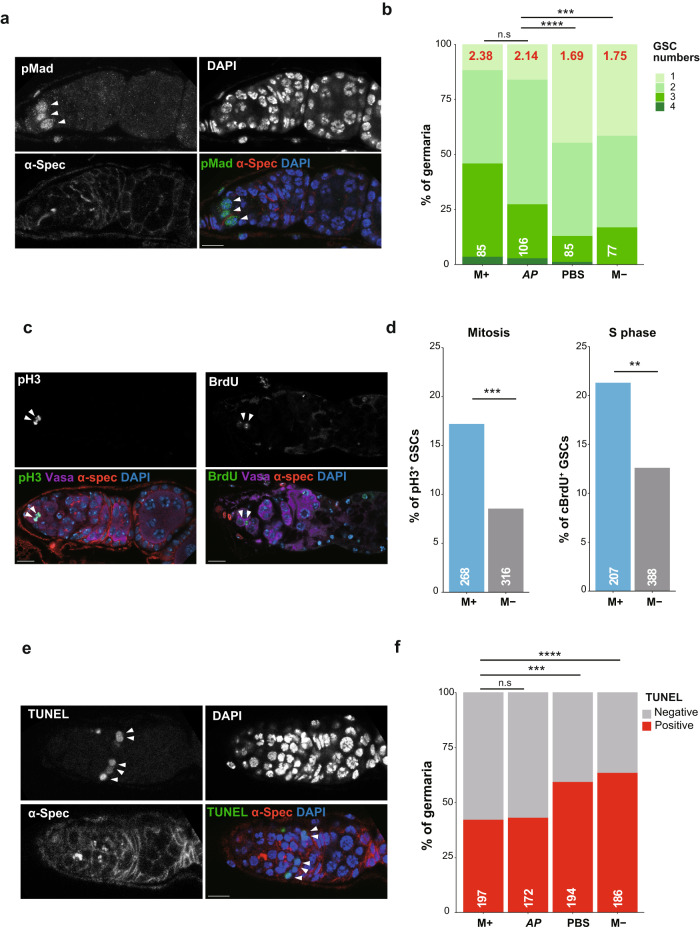
Microbes enhance egg maturation and germline stem cell proliferation. **a** Representative images showing GSCs stained with antibodies against pMad (green), α-Spectrin (α-Spec) (red) and DAPI (blue). GSCs were defined by a GSC marker, pMad, round spectrosome morphology and location (attached to the niche, cap cells). **b** Frequency of germaria containing 1–4 GSCs in females cultured in microbe-rich (M+), microbe-poor (M-), cultured *A. pomorum (AP)* or control (PBS) conditions. The average number of GSCs per germarium and the number of germaria examined are shown on the top (red) and the bottom (white), respectively. **c** Representative images of GSCs stained either with anti-pH3 antibody (left panels) or anti-BrdU antibody (right panels), together with anti-Vas antibody, anti-α-Spec antibody and DAPI (bottom panels). **d** The percentage of GSCs in M phase (positive for pH3) and S phase (positive for BrdU incorporation) in M+ or M- conditions. The number of examined GSCs is shown on the bottom (white). **e** Representative image of germarium containing TUNEL-positive cells (white arrowheads), co-stained for α-Spec (red) a fusome marker, and DAPI (blue). **f** The ratio of germaria containing TUNEL-positive cells for females cultured in M+, *A. pomorum (AP)*, control (PBS) or M- conditions. The number of germaria examined are shown on the bottom (white). For statistical analysis, a Chi-square test is used for (**b, d** and **f**). ****P ≤ 0.001, ***P ≤ 0.005, **P ≤ 0.01, n.s., nonsignificant (*P* > 0.05). Scale bar = 10 μm in (**a, c** and **e**).

*Drosophila* oogenesis in adults is initiated by the asymmetric division of germline stem cells (GSCs) located at the anterior of the germarium^10^. To determine whether microbial sensitization affects GSC proliferation, we quantified GSC numbers by immunostaining with phosphorylated Mad (p-Mad), a GSC marker for the activation of the BMP signaling pathway (Fig. 2a). Consistent with egg numbers, GSCs increased equivalently in females placed in *A.pomorum-*inoculated vials or in microbe-rich (M + ) vials compared to those in the microbe-poor (M-) vials or PBS (Fig. 2b; M+:2.38, AP: 2.14, PBS: 1.68, M−: 1.75 on average). These data indicated that microbes deposited by flies promoted oogenesis by increasing GSC numbers at an early stage of oogenesis (Supplementary Fig. 2f). A comparable increase of GSC number within the germarium was observed for females cultured in vials inoculated with *L.plantarum or L.brevis* (Supplementary Fig. 2e).

To investigate how microbes increase GSC numbers and promote egg maturation, we first examined the mitotic status of GSCs by immunostaining for the M and S phase makers, anti-phospho-histone H3 (pH3) and bromodeoxyuridine (BrdU), respectively (Fig. 2c). Upon inoculation of females in M+ vials, the frequency of GSCs in both M and S phases increased (Fig. 2d; 17.2% and 8.5% for M phase; 21.3% and 12.6% for S phase, in M+ and M−, respectively), suggesting that microbes promoted GSC proliferation by the progression of GSC mitosis in conjunction with an increase of GSC numbers. Next, because nutrition-deprived flies have increased apoptosis of germline cysts in the germarium^13^, we examined the frequency of PCD under microbe-dependent conditions. Defective germline cysts and eggs are eliminated at two checkpoints: at region 2a/b of germarium and mid-oogenesis^13,43–45^. Females cultured in either *A.pomorum-rich* or M+ vials exhibited fewer TUNEL-positive cells, an apoptotic marker detecting DNA fragmentation, compared to females placed in either PBS or M- conditions (Fig. 2e, f). These data suggest that microbes acquired from the environment are capable of increasing GSC numbers through the upregulation of mitotic division and suppression of cell death at a critical checkpoint during oogenesis, thus contributing to enhanced egg development.

### Somatic and germline cell division are accelerated by microbes

**Fig. 3:**
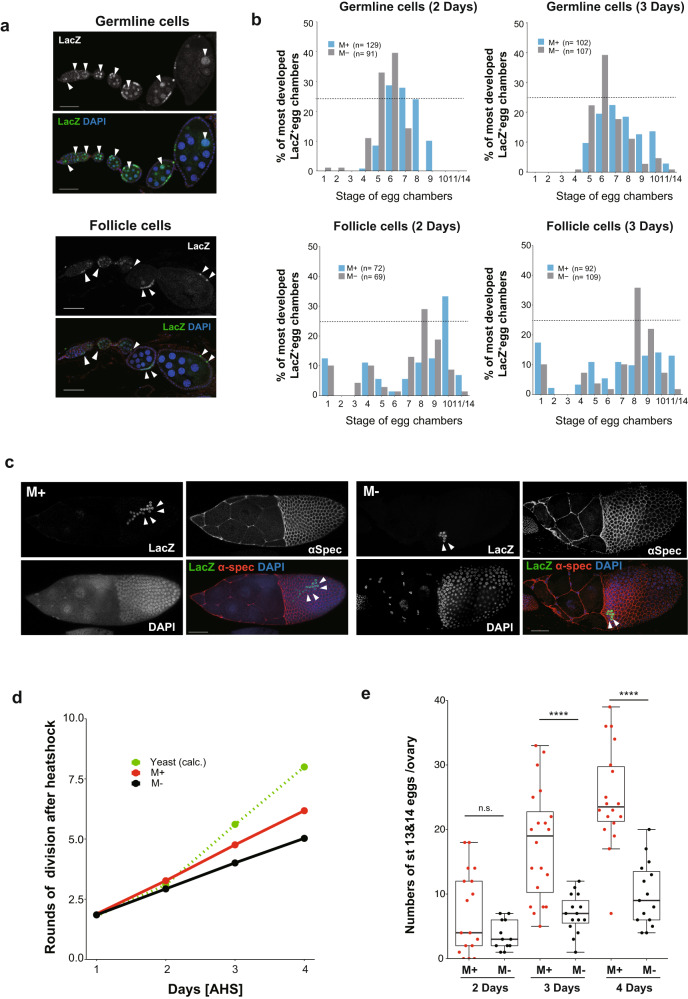
*Drosophila* microbes enhance the progression of oogenesis by acceleration of ovarian cell division. **a** Representative images of heat shock-induced LacZ-positive germline cell clones (upper panels) and follicle cell clones (lower panels) that are continuously derived from stem cells. Ovarioles were stained with anti-LacZ antibody (green, arrow heads) and DAPI (blue). **b** The profiles of the most developed stages of egg chambers containing *LacZ*-positive germline and follicle cell clones in the ovarioles. LacZ-positive cells were counted either at 2 or 3 days after heat shock, and the percentage of the most advanced stages of LacZ-positive cells in the ovarioles were plotted for M+ (blue) or M- (gray) conditions. Dotted line indicates 25% of *y*-axis. The number of egg chambers examined is shown in the graph. **c** Representative images of stage 10 egg chambers containing LacZ-positive follicle clones (arrow heads) in M+ or M- conditions at 3 days after heat shock. Ovaries were stained for *LacZ* (green), α-Spec (red) and DAPI (blue). **d** The number of cell division rounds undergone by follicle cells was measured by counting *LacZ*-positive follicle cells in a clone at stage 10, 2–4 days after heat shock under M+ or M- conditions. The number of cell division rounds in yeast-fed conditions was calculated based on ref. ^20^; (*n* = 5-10 for each point). **e** The number of stage 13/14 eggs per ovary from females cultured in M+ or M− vials at 2, 3, or 4 days after heat shock. For statistical analysis, a Wilcoxon rank sum test is used. ****P ≤ 0.001, n.s., nonsignificant (*P* > 0.05). Data are represented as mean ± standard deviation. Scale bar = 50 μm in (**a**, **c**).

To verify whether microbes influenced ovarian cell division during oogenesis, we investigated the rate of mitotic cell division accompanying developmental growth using a heat shock-induced FRT-LacZ expression system^20,46^. This system allows us to measure the rate of mitotic cell division in germline or somatic cells during oogenesis by visualizing Lac-Z-expressing clones derived from GSCs and follicle stem cells, respectively (Fig. 3a). From two to four days after heat shock, we examined developmental stages of LacZ-positive clones in females containing the FRT-LacZ transgene when placed in M+ or M- conditions. Compared with M- vials, the ovaries of females cultured in M+ vials exhibited continuous LacZ-positive clones of germline and somatic cells in more advanced stages of egg chambers on the second and third days (Fig. 3b). This result suggests that microbes accelerated the proliferation of both germline and somatic cells during oogenesis.

We observed that females cultured in M+ vials possessed more LacZ-positive cells in the clones among epithelial somatic cells in stage 10 egg chambers than those cultured in M- vials (Fig. 3c). We calculated the doubling time of clones based on clone size in stage 10 egg chambers and found that cell division was faster under M+ conditions than M- conditions (Fig. 3d). The division rate is similar to that found under nutrition-rich conditions^20^ (Fig. 3d, 33.7 h vs 45.2 h, in M+ and M−, respectively), suggesting that the M+ condition replicates effects of nutrition-rich conditions on female fecundity. Consistent with the abovementioned results, the egg number of females expressing LacZ cultured in M+ vials was significantly higher than in M- vials (Fig. 3e). These data suggest that the microbiome of flies accelerates the mitotic cell divisions of somatic and germline cells throughout the developmental stages of oogenesis.

### *Drosophila* microbes suppress apoptosis in the germarium

**Fig. 4:**
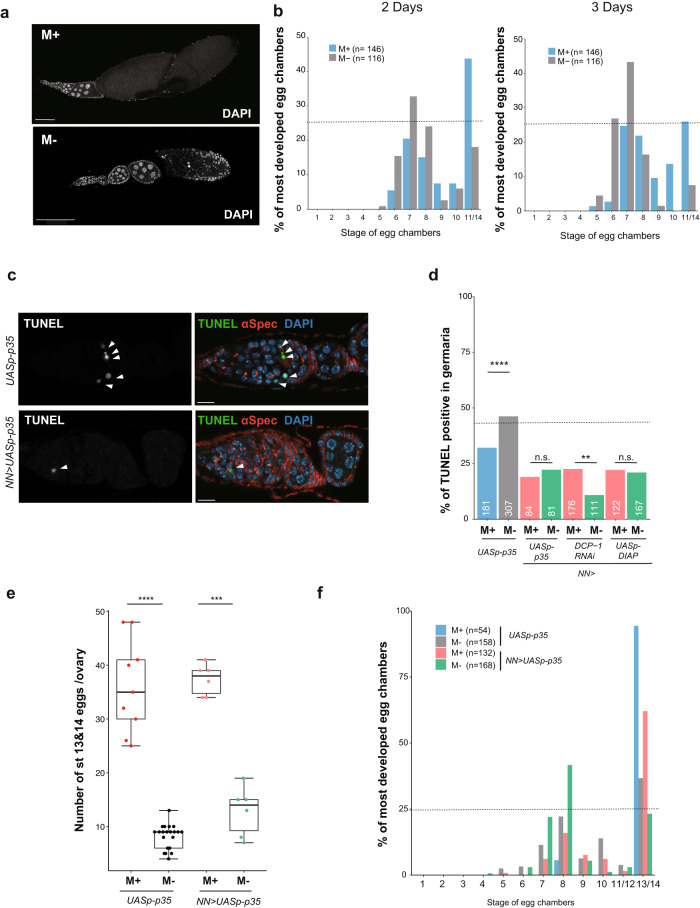
*Drosophila* microbes suppress apoptosis in the germarium but not in mid-oogenesis. **a** Representative images of DAPI-stained egg chambers in females cultured in M+ or M- conditions for 3 days. **b** The profiles of the most developed stages of egg chambers from females cultured in M+ (blue) or M- (gray) vials for 2 or 3 days. **c** Representative images of TUNEL-positive (arrow heads) germaria expressing *UASp-p35* alone or *UASp-p35* driven by *NGT40*; *Nos-Gal4* (*NN-Gal4*) (*NN-Gal4* > *UASp-p35*). **d** The profiles of TUNEL-positive germaria from females of the indicated genotype cultured in M+ or M- vials. Each gene was overexpressed (*p35, DIAP*) or knocked down (*DCP-1*^*RNAi*^) in germline cells by *NN-Gal4*. The number of germaria examined are shown on the bottom (white). **e** The number of stage 13/14 eggs per ovary (*UASp-p35* or *NN-Gal4* > *UASp-p35*) cultured in M+ or M- conditions. For statistical analysis, a Chi-square analysis is used for (**a**) and a Wilcoxon rank sum test is used in (**e**). ****P ≤ 0.001 and ***P ≤ 0.005, **P ≤ 0.01, *n.s.* nonsignificant (*P* > 0.05). Data are represented as mean ± standard deviation. **f** The profiles of the most developed stages of egg chambers (*UASp-p35* or *NN-Gal4* > *UASp-p35*) cultured in M+ (blue or pink) or M- (gray or green) conditions. The number of egg chambers examined are shown in the graph (**b,** **f**). Dotted line indicates 25% (**b** and **f**) or 40% (**d**) of y-axis. Scale bar = 100 μm in (**a**) and 10 μm in (**c**).

Next, we examined the developmental profile of oogenesis by counting each stage of egg chambers on the 2nd and 3rd day of incubation in M+ or M- vials. Ovaries from females cultured in M+ conditions contained more developed egg chambers (stage 11–14) than those in M- conditions (Fig. 4a, b) whereas females in M- conditions had fewer mature eggs and more stage 7–9 egg chambers (Fig. 4a, b). This finding suggests that oogenesis progressed faster with the influence of environmental microbes, while M- conditions hindered oogenesis, possibly by apoptotic cell death at the germarium and mid-oogenesis checkpoint (Fig. 2e, f).

The accumulation of mid-stage egg chambers in females cultured under M- conditions suggests an inability for developing eggs to overcome the second checkpoint around the mid-stage. To clarify the involvement of PCD specifically at the second checkpoint, we suppressed cell death by manipulating gene functions related to apoptotic events or autophagy. Expression of an anti-apoptotic protein p35 in germline cells suppressed cell death in the germarium of females cultured in M+ or M- vials to a similar degree (Fig. 4c, d). Apoptosis in the germarium was also repressed by germline knockdown of *Death caspase-1 (DCP-1)* and overexpression of *Death-associated inhibitor of apoptosis 1* (*DIAP1*), but not by knockdown of *Atg1*, an autophagy-regulated gene (Fig. 4d, Supplementary Fig. 3a, g, h). Altogether, these results suggest that germline cells are primarily eliminated in an apoptosis-dependent manner, a process that is suppressed by microbes in the germarium through a caspase-dependent pathway.

We then examined whether suppressing apoptotic events in the germarium would enhance mature egg production. Surprisingly, egg numbers in females that have suppression of apoptosis by p35 or *DIAP1* overexpression or *DCP-1* knockdown under M- conditions were still fewer than in those under M+ conditions (Fig. 4e, Supplementary Fig. 3b-f). Instead, disruption of cell death pathways in females cultured in M- vials led to more mid-staged eggs than females cultured in M+ vials, despite the suppression of apoptosis in the germarium (Fig. 4d, f, Supplementary Fig. 3b–f). In contrast, neither overexpression of *p35* or *DIAP1* nor knockdown of *DCP-1* or *Atg1* involved in apoptosis or autophagy by a somatic driver *(tj-Gal4*) increased egg number or suppressed cell death in the germarium under M- conditions (Supplementary Fig. 3i, j), suggesting that somatic inhibition of apoptosis does not play a role in cell death during oogenesis. Taken together, these results suggest that the second apoptotic checkpoint is controlled by caspase-independent pathways in germline cells and that acquired microbes facilitate progression through each checkpoint to achieve egg maturation.

### Ecdysone pathway controls egg maturation and GSC numbers

**Fig. 5:**
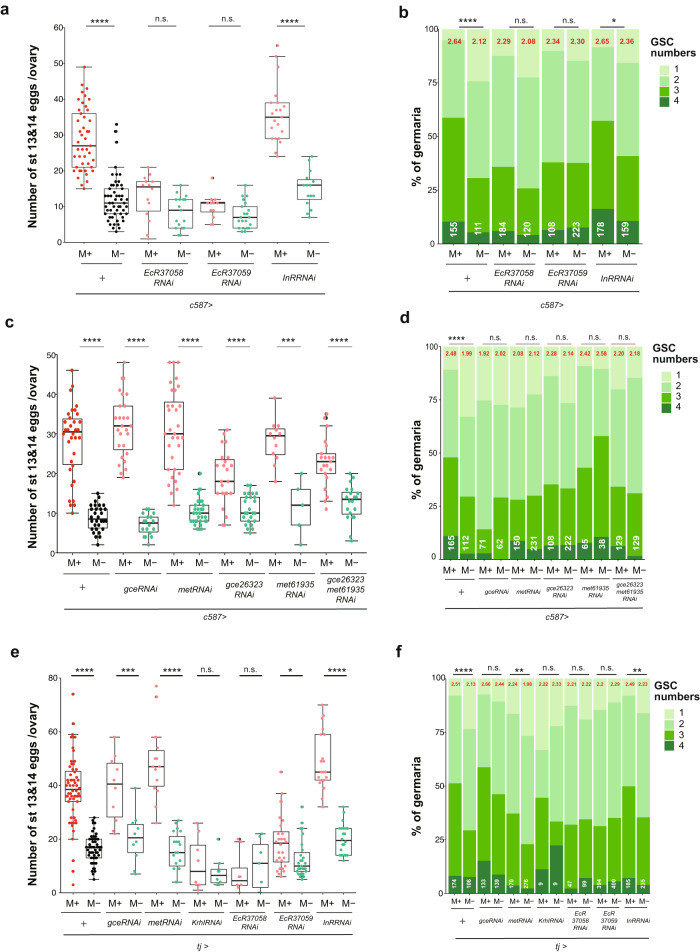
The ecdysone pathway controls the egg maturation and GSC numbers, while the JH pathway is involved only in GSC proliferation. **a, c, e** The number of stage 13/14 egg chambers per ovary of females of the indicated genotype cultured in M+ or M- vials. **b, d, f** Frequency of germaria containing 1–4 GSCs of females cultured in M+ or M- vials. pMad-positive cells located next to cap cells are counted as GSCs. The average number of GSCs per germarium and number of germaria examined are shown on the top (red) and the bottom (white), respectively. **a**, **b** Ecdysone or insulin pathway-related genes were knocked down (*EcR37058*^*RNAi*^, *EcR37059*^*RNAi*^ *or InR*^*RNAi*^) using the somatic driver, *c587-Gal4*. **c**, **d** Juvenile hormone pathway-related genes (*gce*^*RNAi*^, *met*^*RNAi*^*, gce26323*^*RNAi*^*, met61935*^*RNAi*^ or *gce26323*^*RNAi*^ */met61935*^*RNAi*^) were knocked down by *c587-Gal4*. **e**, **f** Juvenile hormone, ecdysone or Insulin pathway-related genes (*gce*^*RNAi*^, *met*^*RNAi*^*, kr-h1*^*RNAi*^*, EcR37058*^*RNAi*^, *EcR37059*^*RNAi*^ or *InR*^*RNAi*^) were knocked down using the somatic driver *tj-Gal4*. For statistical analysis, a Wilcoxon rank sum test is used for (**a, c** and **e**) and a Chi-square analysis is used for (**b**, **d** and **f**). ****P ≤ 0.001, ***P ≤ 0.005, **P ≤ 0.01, *P ≤ 0.05, n.s., nonsignificant (*P* > 0.05). Data are represented as mean ± standard deviation for (**a, c**, and **e**).

Hormones, such as insulin, ecdysone, and juvenile hormone, regulate *Drosophila* oogenesis by modulating GSC number, oogenesis progression, and suppression of apoptotic cell death^30,31,47–50^. Our results indicate that microbes promote oogenesis by contributing to each of these processes. These multi-tiered regulations are reminiscent of the versatile effects of hormones on oogenesis development. Therefore, we examined the involvement of hormonal pathways as a mechanism by which environmental microbes enhance oogenesis. First, we used the *c587-Gal4* driver expressed in cap cells and escort cells in the germarium^51^ to suppress hormone activity by shRNA in ovarian somatic tissues and evaluated the effects on egg and GSC numbers. We found that the perturbation of ecdysone receptors (EcRs) significantly reduced egg numbers under M+ conditions to a level similar to that of females in M- conditions (Fig. 5a), suggesting that the ecdysone pathway is necessary for the microbe-induced increase in mature egg number. In striking contrast, disruption of the receptor for insulin-like peptides, a well-known pathway that regulates physiological responses to nutrition, did not affect the number of mature eggs induced by M+ conditions, which was comparable to that of wild-type female flies. To further confirm this finding, we reduced the amount of insulin-like peptides *IIp2 or IIp3* by expressing the pro-apoptotic *rpr* gene in insulin-producing cells and found negligible effects on microbe-enhanced egg maturation (Supplementary Fig. 4a). Finally, female mutants defective in *Ilp2*, *3*, *5*, and *6* production^15,23^ also showed increased egg numbers under M+ conditions. In short, none of the examined insulin-related pathway genes appeared to be necessary for microbe-enhanced egg maturation (Supplementary Fig. 4a). Similarly, the microbe-induced increase of GSC numbers was canceled by perturbation of EcR, but not InR (Fig. 5b), suggesting GSC proliferation is regulated by ecdysone pathway. These results strongly suggest that ecdysone signaling, but not insulin, is a primary hormone pathway necessary for microbe-enhanced oogenesis.

We examined a second major hormone pathway in *Drosophila* oogenesis, Juvenile hormone (JH). JH activity is transduced through partially redundant receptors Germ cell-expressed (Gce) and Methoprene-tolerant (Met) and the downstream receptor, Krh1, which can function cooperatively or independently^52–54^. Egg numbers under M+ conditions did not change upon disruption of *gce* and/or *met* expression by the *c587-Gal4* driver (Fig. 5c). It was difficult to evaluate the contribution of *kr-h1* since disruption of the gene results in severe defects in ovarian development (Supplementary Fig. 4b, c). In contrast, the effect of microbes on enhancing GSC numbers was canceled upon suppressing expression of JH receptor genes (*gce*, *met*) individually or simultaneously (Fig. 5d). We found that microbes, in addition to knockdown of hormone receptors in the ovarian somatic cells, did not affect the number of cap cells, a main niche component, indicating that microbe-induced GSC proliferation as well as knockdown of hormone receptors is achieved without disturbing niche architecture (Supplementary Fig. 4d), as well as the morphology of germarium. Each shRNA was capable of reducing the expression of target gene as confirmed by qPCR following knockdown in the salivary gland (*Sgs3-Gal4*, Supplementary Fig. 4e).

To rule out the possibility that there may be insufficient receptor knockdown by *c58*7-*Gal4* in late follicle cells, another somatic driver, *tj-Gal4*, which is expressed in the early and later stages^51^, was also used to inhibit the expression of the hormone receptors. Similar to the results of *c587-Gal4*, abrogation of EcR expression by *tj-Gal4* abolished microbe-enhanced egg production and increased GSC numbers in the M+ condition, suggesting that the ecdysone pathway is essential in microbe-enhanced oogenesis (Fig. 5e, f). As with *c587-Gal4*, InR knockdown by *tj-Gal4* did not cancel the enhancement effect on both GSC numbers and mature egg production in females placed in M+ vials (Fig. 5e, f), providing further evidence that insulin-like peptides are dispensable for microbe-induced oogenesis. Moreover, disruption of JH signaling by knockdown of JH receptors with *tj-Gal4* or inhibiting JH production by overexpression of *Nuclear Inhibitor of Protein Phosphatase (NiPp)* also did not change egg numbers observed under M+ conditions (Fig. 5e, Supplementary Fig. 4a). As such, JH receptors do not appear to be relevant in egg maturation despite influencing GSC numbers (Fig. 5e, f). Although both *tj-Gal4* and *c587-Gal4* are reported to have expression in the brain^51^, pan-neuronal RNAi knockdown of hormone receptors by *elav-Gal4* did not alter the microbe-induced increase in GSC number or egg maturation (Supplementary Fig. 5a, b), confirming that hormonal signaling in the ovaries regulates microbe-induced oogenesis. Furthermore, disruption of hormone receptors in germline cells by the *Nanos-Gal4; NGT40* driver did not affect microbe-induced GSC numbers and egg maturation (Supplementary Fig. 5c, d). This observation suggest that somatic hormone receptors function similarly in microbe-induced oogenesis and mating-induced oogenesis^24^. Altogether, our results support the following model: microbes promote egg maturation predominantly via the ecdysone pathway, while promoting GSC proliferation through both ecdysone and juvenile hormone pathways.

### Excessive 20E and JH promote the proliferation of GSCs

**Fig. 6:**
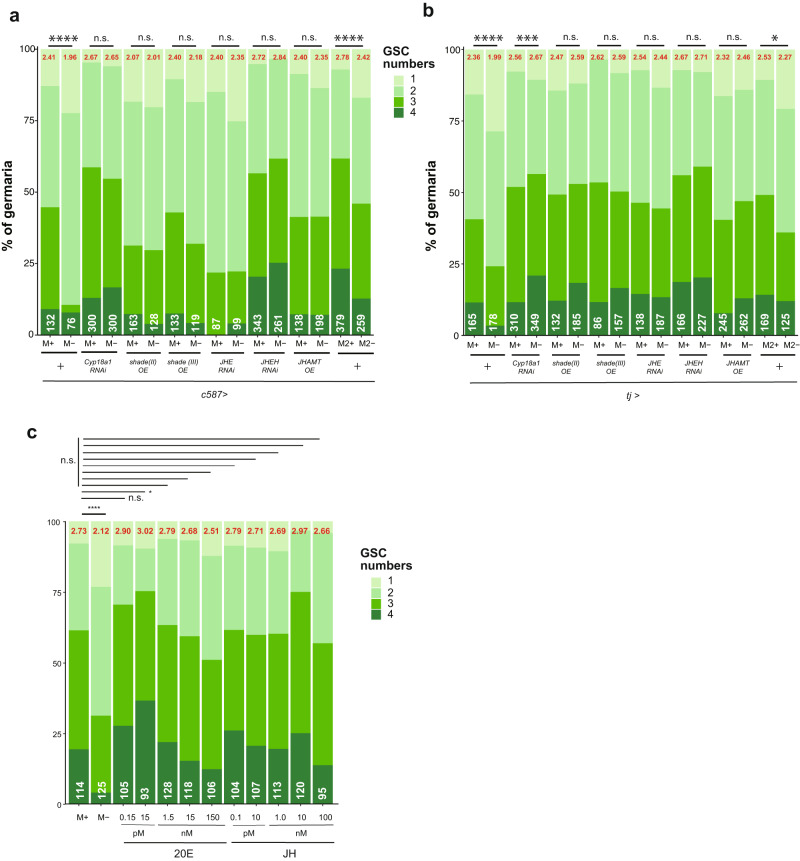
Excessive 20E and JH increase the number of GSCs. **a, b** Frequency of germaria containing 1-4 GSCs from females of the indicated genotypes cultured in M+ or M- vials. pMad-positive cells found next to cap cells were counted as GSCs. The average number of GSCs per germarium and number of germaria examined are shown on the top (red) and the bottom (white), respectively. The enzymes involved in 20E biosynthesis were knocked down (*Cyp18*^*RNAi*^*)* or over-expressed (*shade(II) OE* or *shade(III) OE)* using *c587-Gal4* in (**a**), or *tj-Gal4* in (**b**). The enzymes involved in JH biosynthesis were knocked down (*JHE*^*RNAi*^ or *JHEH*^*RNAi*^) or over-expressed (*JHAMT OE)* using *c587*-*Gal4* in (**a**), or *tj-Gal4* in (**b**). **c** Frequency of germaria containing 1–4 GSCs in the ovaries cultured ex vivo in the presence of synthetic 20E or JH at the indicated concentrations. For statistical analysis, a Chi-square analysis is used. ****P ≤ 0.001, ***P ≤ 0.005, *P ≤ 0.05, n.s., nonsignificant (*P* > 0.05).

To further confirm that ecdysone and juvenile hormone pathways activate oogenesis in a M+ environment, we manipulated the amount of each hormone ligand by knockdown or overexpression of enzymes involved in 20E or JH biosynthesis in ovarian somatic cells with either *c587-Gal4* or *tj-Gal4*^55–57^. 20E biosynthesis requires cytochrome p450-encoding genes *shade* whereas its inactivation is partially mediated by *Cyp18a1*^58–60^. Hence, overexpression of *shade* or knockdown of *Cyp18a1* (Supplementary Fig. 6a, b) is expected to increase 20E levels in ovarian somatic cells. These manipulations increased the frequency of GSCs in the germarium despite being under M- conditions, similar to those under M+ conditions (Fig. 6a, b). In contrast, egg production was unchanged under M- conditions when *tj-Gal4* was used to manipulate *Cyp18a1* or *shade* expression (Supplementary Fig. 6d). Unexpectedly, overexpression of *shade* using *c587-Gal4* resulted in a slight reduction of mature eggs even under M+ conditions (Supplementary Fig. 6c).

To increase JH levels, we knocked down genes encoding inactivation enzymes, *Juvenile hormone esterase* (*JHE*) and *Juvenile hormone epoxide hydrolase* (*JHEH*), or overexpressed a key biosynthesis component, *Juvenile hormone acid methyltransferase* (*JHAMT*) by either *c587-Gal4* or *tj-Gal4* (Supplementary Fig. 6a, b). Upregulation of JH by these somatic drivers, as with 20E, allowed GSCs to proliferate under M- conditions (Fig. 6a, b) although egg production in these females did not increase (Supplementary Fig. 6d). However, females with *JHE* knockdown and *JHAMT* overexpression driven by c58*7-Gal4* exhibited reduced numbers of mature eggs under M+ conditions (Supplementary Fig. 6c). The unexpected suppression of egg production in these females could be due to excess levels of the hormone in early somatic cells causing negative feedback in the pathways for egg development. The lack of increased egg maturation upon manipulating the biosynthesis of hormone ligands may be due to insufficient numbers of receptors to which excess ligand could bind^59,61–63^; however, we could not measure the precise hormone levels by the reporter systems due to the technical limitations. We, therefore, further examined GSCs after culturing ovaries ex vivo in the presence of synthetic 20E or JHIII. Ovaries cultured with the hormone ligands possessed more GSCs than control, a similar number to those under M+ condition (Fig. 6c), indicating that 20E or JH directly influences the ovaries in controlling GSC numbers. Overall, these results suggest that 20E and JH are necessary for microbe-mediated GSC proliferation.

### Microbes regulate hormonal pathways during oogenesis

**Fig. 7:**
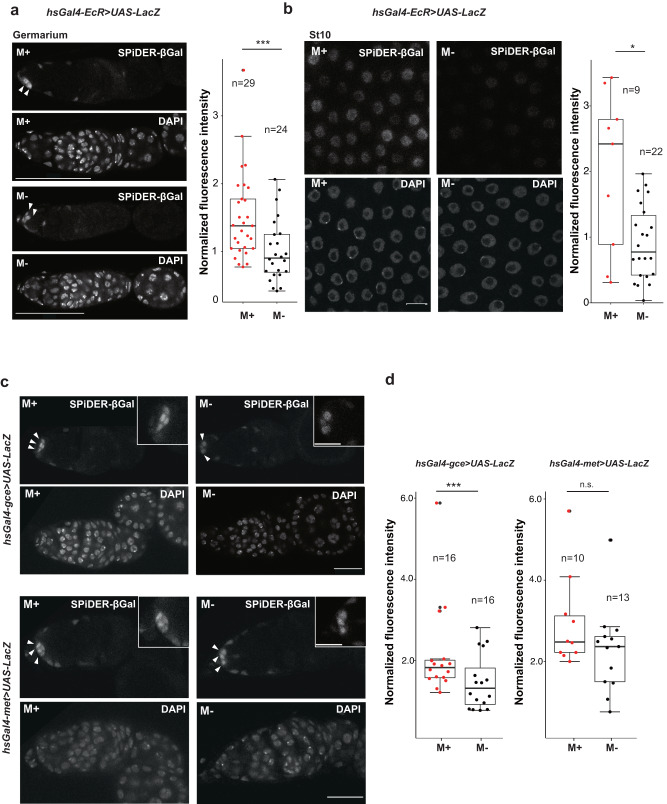
Microbes activate hormonal pathways during oogenesis. Representative images showing activation of EcR by LacZ expression in the germarium (**a**) and follicle cells in stage 10 egg chambers (**b**) from females cultured in M+ or M- conditions (left). Females containing *hs-Gal4-EcR* > *UAS-LacZ* were heat shocked and dissected for histochemical staining of ovaries by SPiDER-βGal and DAPI. The fluorescence intensity of SPiDER-βGal (514 nm) is normalized to that of DAPI signal (405 nm) (right). **c** Representative images showing activation of *gce* or *met* by LacZ expression in germaria from females cultured in M+ or M- conditions. Females containing *hs-Gal4-gce* > *UAS-LacZ* (Top) or *hs-Gal4-met* > *UAS-LacZ* (Bottom) were heat shocked and dissected for histochemical staining of ovaries by SPiDER-βGal and DAPI. **d** The fluorescence intensity of SPiDER-βGal is normalized to that of DAPI signal. For statistical analysis, a Wilcoxon rank sum test is used for (**a, b**, **d**). ***P ≤ 0.005, *P ≤ 0.05, n.s., nonsignificant (*P* > 0.05). Data are represented as mean ± standard deviation. Scale bar = 50 μm in (**a**), 10 μm in (**b**) and insets of **c** and 20 μm in (**c**).

Based on the outcomes of the ex vivo experiments, we next examined whether the presence of environmental microbes led to the activation of ecdysone or JH receptors. We utilized the heat shock-induced *EcR-LacZ* sensor system and visualized receptor activation of LacZ by the chemically-modified fluorescent reagent SPiDER-βGal^64,65^. Activation of the sensor was initially confirmed with the synthetic steroid compound Ponasterone A (PonA) (Supplementary Fig. 7a). The intensity of LacZ in the ovaries, anterior somatic cells in germarium and stage 10 of the egg chambers, from females bearing the *EcR-LacZ* transgene under M+ conditions has stronger than those under M- conditions (Fig. 7a, b). Our results indicated that environmental microbes increased EcR activation in ovarian somatic cells both in early and late stages. Indeed, we measured 20E amounts using enzyme immunoassay (EIA)^24^ and observed more 20E under M+ conditions when there is greater ovarian tissue mass. The same trend was observed after normalization of the respective weight of the ovaries, though the difference was not statistically significant (Supplementary Fig. 6e). These results imply that increased 20E levels under M+ conditions contribute to the activation of ovarian EcR.

We further examined the activation of JH receptors using newly generated transgenic flies, *hsGal4-gce* and *hsGal4-met*, traced with *UAS-LacZ*. The reporter system was initially validated with synthetic JHIII (Supplementary Fig. 7b). The JH receptor Gce but not Met, had stronger fluorescence intensity in anterior somatic cells in germaria under M+ conditions than M- conditions, suggesting that Gce was activated in the early somatic cells by microbes (Fig. 7c, d). In contrast, there were no differences in the fluorescence signal of LacZ for either receptor at mid-late stage, indicating that JH signaling is not involved in the later stages of egg development (Supplementary Fig. 7c). In conclusion, microbe-induced activation of the ecdysone pathway in early somatic cells and later stages of follicle cells promotes GSC proliferation and egg maturation. In contrast, the JH receptor Gce, but not Met, was activated in the germarium in response to microbes, suggesting that JH signaling via Gce primarily functions in GSC proliferation.

## Discussion

Our study revealed that environmental microbes promote GSC proliferation and ovarian germline and follicular cell division, while suppressing apoptosis at two developmental checkpoints, leading to increased egg production. The following lines of evidence support the role of microbes in promoting oogenesis: (1) germ-free flies do not accelerate egg maturation (Fig. 1c) nor increase GSC numbers (Supplementary Fig. 2f), (2) pheromone extracts or the lack of pheromone-responsive or chemo-sensitive receptors had no discernable effect on oogenesis (Supplementary Fig. 1g, h), and (3) Heat-inactivated microbe strains or swab-wash eluate from donor-sensitized vials abolished oogenesis enhancement properties (Fig. 1g, Supplementary Fig. 1e), thus implying that heat-labile metabolic substances cause this enhancement^66,67^.

Laboratory-reared *D. melanogaster* has relatively simple microbial diversity comprising 5–20 taxa, with *A.pomorum* and *L.plantarum* being the most abundant species^68,69^. Notably, in our study, regardless of the relative proportions of the microbes^42^, oogenesis was accelerated to a similar extent when females were cultured in the vials treated with *A. pomorum, L. plantarum* or laboratory-reared flies (Figs. 1g, 2b, Supplementary Fig. 2d, e). Consistent with previous studies^68–70^, our results indicated that female flies acquired a similar microbiome profile to that of the donors (Fig. 1d–f). Indeed, some studies have reported that microbiome compositions can change over time and between generations to increase host fecundity in the face of nutritional deficiency, a phenomenon considered an evolutionary adaptation of microbes in specific circumstances^71,72^. Our results may represent the initial phase of adaptation during which environmental microbes improve host reproduction. These findings are consistent with previous studies showing that a combination of intrinsic microbial functions can change host fitness and control reproduction, development, and longevity^7^. Thus, plasticity of the microbiome composition could facilitate rapid host adaptation to different environmental conditions by modulating female fecundity.

A nutrition-rich diet can enhance the mitotic division of GSCs and follicle cells, resulting in an increased production of mature eggs via the insulin pathway^23^. Recently, mating stimuli were shown to increase mature eggs and GSCs by activating GSC mitotic division through the ecdysone pathway^24,73^. In our study, we revealed that environmental microbes deposited by individual flies or cultured *Acetobacter* and/or *Lactobacillus* were able to enhance oogenesis only under poor nutrition conditions (1% yeast), comparable to the level reported for nutrition-rich conditions (5% yeast) as mentioned above. We further found that microbes accelerated the mitotic division of germline and follicle cells, albeit at a slightly slower rate than under nutrition-rich conditions (Fig. 3d)^20^. In conclusion, microbes can compensate for the lack of nutrition in a nutrition-deficient environment and enhance host fecundity^4,20^. Interestingly, the knockdown of apoptosis pathway-related genes under poor nutrition conditions suppressed both microbe-independent and microbe-dependent apoptosis in the germarium but not in the mid-oogenesis stages. One possible explanation is that the checkpoint in mid-oogenesis is largely affected by other signaling pathways or environmental cues^43^. In contrast, under M- conditions, perturbation of the autophagy pathway did not affect PCD at either checkpoint, indicating that PCD at the early checkpoint was regulated by apoptotic events rather than autophagy. Interestingly, PCD resulting from the complete starvation of amino acids or in response to mating was suppressed by the knockdown of autophagy pathway-related genes (*Atg1*)^13,74,75^, suggesting a different regulatory mechanism for PCD in response to microbes. Taken together, under poor nutrition conditions, microbe-derived metabolites suppress PCD at the second checkpoint through hormonal regulation or pathways other than apoptosis or autophagy, thereby promoting oogenesis beyond the vitellogenesis stage.

Hormonal pathways, such as ecdysone, insulin, and juvenile hormones, regulate multiple critical processes of *Drosophila* oogenesis: GSC proliferation, apoptosis at two checkpoints, progression of vitellogenesis, and lipid accumulation in oocytes^19,20^. Surprisingly, none of the nutrition-responsive canonical pathways including insulin signaling, amino acid transporters^76^, or regulation of adipokinetic hormone^77^ played a role in microbe-enhanced oogenesis (Fig. 5, Supplementary Fig. 4a). Instead, microbes enhanced GSC proliferation through both the ecdysone and JH signaling pathways but promoted egg maturation only via ecdysone. We observed that knockdown of the somatically-expressed EcR increased the number of TUNEL-positive germarium in the M+ condition, similar to the M- condition, suggesting that ecdysone signaling regulates PCD in the early stages of oogenesis, in addition to accelerating egg maturation (Supplementary Fig. 4f). This observation is supported by the previous studies that EcR regulates PCD in the other tissues^78,79^. In previous studies, ecdysone signaling is also required for proliferating female GSCs in response to mating and during the aging process in *Drosophila* without a change in cap cell numbers^24,80,81^. Thus, the microbe-induced process employs somatic receptors in promoting GSC proliferation non-cell autonomously in a similar way to mating but distinct from the aging process that requires ecdysone signaling in GSCs^30^ (Supplementary Fig. 4d, 5c, d).

Unlike the activation of EcRs in the germarium and stage 10, the JH receptor Gce, but not Met, was activated only in the germarium (Fig. 7)^31^. This result raises the possibility that the two JH receptors differ in their affinity for JH ligands or may sequentially bind to the ligands in vivo. Consistent with our observation that the JH receptors were activated to an equivalent degree under M+ or M- conditions at later stages of oogenesis (Supplementary Fig. 7c), we observed similar levels of the hormone in the hemolymph of females under M+ and M- conditions (Supplementary Fig. 7d). Circulating JH levels correlate with egg maturation in response to mating^82^ or the expression of yolk protein precursors during vitellogenesis in other insects^53^. These results are consistent with our findings that JH does not appear to play a role in egg maturation during microbe-induced oogenesis (Fig. 5c, e). Accordingly, microbes activate EcR and JH hormonal pathways separately or interdependently at an earlier stage of oogenesis, whereas ecdysone pathways primarily control the vitellogenic stage. However, the underlying molecular mechanisms and tissue sites at which the microbial regulation of hormones takes place remain elusive.

In flies, metabolites produced by gut microbes are known to impart a systemic influence throughout the body and can help to maintain host physiological homeostasis. For example, short-chain fatty acids, which are byproducts of bacterial fermentation, modulate gut peptide hormone secretion, insulin release from pancreatic cells, appetite control, and energy homeostasis^36,83,84^. Citrate, a gut-derived metabolite, promotes sperm maturation in the adjacent testes, and increases food intake^85^. Vitamins provided by microbes indeed activate mitochondria function and enhance host energy and reproduction^5^, although under our conditions, vitamins are not as effective as M+ conditions in enhancing host reproductivity (Supplementary Fig. 7e, f). Similarly, mating remotely activates fly oogenesis through inter-organ communication^24,73,86^. Therefore, identifying microbe-induced molecular processes and circulating metabolites exchanged between host tissues will likely uncover cellular processes intrinsically tied to reproduction. The novel findings in host-microbe interactions can open new horizons to improve reproductive health, possibly through probiotic treatments.

## Materials and methods

### *Drosophila* strains

All stocks were maintained at 25°C on standard cornmeal fly food. CS9515 flies were used as wildtype (WT). The fly strains used in the study are listed in Supplementary Table 2.

### Generation of hs-GAL4-LBD transgenic fly lines

The yeast GAL4 DNA-binding domain (amino acid 1-147) was amplified by PCR from fly extract containing the Gal4 transgene using primers; GAL4_FW and GAL4_RV. p-attB-FL.hsp70P-Dam [4-HT-intein@L127C] Myc [open] (Addgene #71805) was amplified with the region (1-485, 516-8446) with the primers; VFW and VRV. LBD domain of Gce (amino acid 342-959) was amplified with Gce_Fw and GAL4_GceRV.  The LBD domain of Met (amino acid 104-716) was amplified with Met_Fw and GAL4_Met_RV. hs-GAL4-Gce or Met was introduced for transgenic fly lines. Integrase-mediated transformation procedures introduced each construct into the germline of attp40 flies. The homozygous viable transgenic lines carrying hs-GAL4-Gce or Met on the second chromosome were isolated and used for all studies reported here. Primers are listed in Supplementary Table 3.

### *Drosophila* microbe-sensitization assays

To generate sensitized or microbe-rich (M+) conditions, five male pupae were placed into fresh food vials. After 3–4 days, the males were removed and replaced with five female pupae. For unsensitized or microbe-poor (M−) conditions, no male pupae were placed in the vials. Female pupae were cultured for 3 days prior to the evaluation of oogenesis. Three vials of M+ and M- vials were set up for each experiment. For egg counting assays, ovaries from individual female flies were dissected and stage 13 and 14 eggs were counted when mounting ovaries on glass slides. GSC numbers were counted using anti-pMad staining (Described below). Low yeast food (corn flour 7%, yeast 1%, sucrose 2%, dextrose 5%, agar 1%, Nipagen 10%) was used for all microbial assays. For statistical analysis, the Wilcoxon rank sum test is used for egg numbers. A Chi-square test is used for GSC numbers. (****P ≤ 0.001, ***P ≤ 0.005, **P ≤ 0.01, *P ≤ 0.05, n.s., nonsignificant (*P* > 0.05)).

To sensitize vials with swabbed inoculant, male pupae were first placed in fresh food vials. After 3 days, the eclosed flies were removed and the wall of the sensitized vial was swabbed for 30 s with a sterile swab. Next, the swab was soaked in 200 μL of PBS and gently agitated. The PBS wash was added to a fresh vial and female pupae were added after 3 days. Heat inactivation of the PBS wash was performed at 65 °C for 10 min. To sensitize vials with male pheromones, pheromone extract was first prepared by placing five males in a glass vial with 200 μL hexane. After 10 min at RT, the hexane extract was transferred to a fresh food vial and allowed to evaporate. Hexane alone was added to control vials as a solvent control. We evaluated the effects on individual females from three vials in each condition because deviation among individuals was lower than the average of vials.

### Counting colony forming units (CFUs) from donor or recipient flies

To measure the microbial load in the whole fly body based on CFU counts, we followed established protocols^41^ with some modifications. After homogenizing flies in 125 μL of PBS with sterile pestles for 1 min, the homogenate was diluted to 1 mL. 10 μL of serial dilutions at 1:9, 1:81, and 1:729 was applied to the mannitol and MRS plates. The plates were incubated at 30 °C for 2 days, and the number of colonies was counted.

### Extraction of microbe genome

Fly surfaces were sterilized by washing twice in 95% EtOH followed by washing twice in sterile water. Single flies were placed in tubes with 1.4 mm ceramic beads (Qiagen; MD, USA) and ATL buffer from PowerMag Bead Solution (Qiagen) and homogenized with a bead mill homogenizer (Bead Ruptor Elite, Omni, Inc; GA, USA), following extended vortex for 45 min at 4 °C. Following an overnight proteinase K treatment (2 mg/mL) at 56 °C, DNA was extracted using the MagAttract PowerSoil DNA EP Kit (Qiagen) according to the manufacturer’s instructions. 16S rRNA gene of bacteria was identified by PCR amplification with primers to the V3-V4 region (515F: GTGYCAGCMGCCGCGGTAA; 806R: GGACTACNVGGGTWTCTAAT)^87^. The primers contain a 12-base pair Golay-indexed code for demultiplexing. PCR reactions were performed with the KAPA3G Plant kit (Sigma Aldrich, MO, USA) under the following conditions: 95 °C for 3 min, followed by 35 cycles of 95 °C for 20 s, 50 °C for 15 s, 72 °C for 30 s, and a final extension for 72 °C for 3 min. The PCR products were purified and normalized using the Just-a-plate kit (Charm Biotech, MO, USA). High throughput sequencing (HTS) was performed with Illumina MiSeq and 250 bp paired-end kits (Illumina, Inc., CA, USA).

### 16S amplicon analysis

FASTQ files were processed using the MetaFlow|mics analysis pipeline^88^ for filtering, denoising, and merging. Contigs were clustered into OTUs with 100 (ASVs) thresholds having consensus classification. We remove all chloroplasts, mitochondrial DNA, and contigs that do not annotate at the kingdom level. For subsampling, the threshold is chosen as the 10th percentile of sample sizes, about 20k and samples with a size below this threshold are discarded. Co-occurrence patterns were corrected with LULU^89^. LULU’s goal is to correct erroneous OTUs that are likely due to PCR/sequencing errors. LULU merges contigs with high similarity (97% in our case) that systematically co-occur in the same samples, one contig being always more abundant than the other. Finally, we removed any OTU with a total abundance of below three. Additionally, we compute the relative abundance tables, the weighted Unifrac distances, and some summary metrics using MOTHUR^90^. Based on the results of the OTU count of each condition, phyloseq (R: v3.63) was used for visualizing the abundance of microbes and the evaluation of the diversity of the variance of microbes in the samples.

### Microbial culture

The isolated microbes (*Acetobacter pomorum*, *Lactobacillus brevis EW,* and *Lactobacillus plantarum WJL)* were grown on the appropriate plates (*Ap*: mannitol, *Lb, Lp*: MRS) in either aerobic (*Ap)* or anaerobic conditions (*Lb, Lp*) at 30 °C. The Anaero Pack Pouch (MITSUBISHI GAS CHEMICAL) was used to generate an anaerobic environment. After inoculating the appropriate broth for each microbe, the cells were washed with PBS three times. The microbe pellet was suspended in PBS, placed on the food in a fresh vial, and incubated at 25 °C for 3 days prior to adding female pupae.

### Germ-free flies

Germ-free flies were generated following established protocols^41,91^. In detail, the embryo was dechorionated in 2.5% sodium hypochlorite in 0.05%Tween 20 in PBS (PBT) for 2 min. Embryos were subsequently washed three times in PBT, followed by washing with PBS three times. The embryos were maintained in vials with axenic food that contained antibiotics (40 μg/mL ampicillin, 100 μg/mL tetracycline, 8.3 μg/mL erythromycin, 40 μg/mL chloramphenicol). The presence of bacteria was checked using genomic PCR analysis and plating on agar medium from fly homogenates. Three whole flies were lysed in lysis buffer (ATL, QIAamp DNA Micro Kit, QIAGEN), sonicated by beads (Pathogen Lysis Tubes L, QIAGEN) for 45 min at 4 °C and purified using QIAamp DNA Micro Kit after overnight incubation at 56 °C. 16S rRNA primers (8FE: AGAGTTTGATCCTGGCTCAG and 519R:GWATTACCGCGGCKGCTG, 341F:CCTACGGGNGGCWGCAG and 805R:GACTACHVGGGTATCTAATCC) were used for genomic PCR. For plating, three flies were homogenized in PBS and the homogenate was diluted 1:10, applied to MRS or mannitol medium, and incubated for 2–3 days at 30 °C.

### Quantitative reverse transcription PCR

After extraction of tRNA by Trizol (Thermo Fisher Scientific) from either ovaries or salivary glands, reverse transcription was performed using the SuperScript III (Thermo Fisher Scientific) with 1 μg of total RNA. qRT-PCR was performed on a Step One Plus Real-Time PCR (Thermo Fisher Scientific) in biological triplicates. The expression of targets in the samples was quantified based on the ddCT method (Livak and Schmittgen, 2001). Fold-change was calculated in comparison with rp49. All primers are listed in Supplementary Table 3.

### Immunostaining

For immunostaining, ovaries were dissected and fixed in 5.3% PFA in PBS for 10 min and washed three times for 20 min in 0.2% TritonX-100 in PBS (PBX). After incubating with blocking buffer (4% BSA, 0.2% PBX) for 40 min, ovaries were incubated with primary antibodies (Supplementary Table 2). overnight at 4 °C followed by three washes in PBT for 15 min each. Incubation with Alexa Fluor-secondary antibodies diluted in 0.4% BSA in PBS was carried out overnight at 4 °C followed by three washes in PBX for 15 min each. Nuclei were stained with DAPI (1:500 in wash buffer). Samples were mounted in mounting media (Fluoro-KEEPER, Nacalai). The images were obtained with a Zeiss LSM900 or LSM780 under ×63 or ×40 magnification. GSC counting was performed with an Olympus Axiovert. Cap cell counting was performed with a Zeiss LSM900. A chi-square test was used to compare pH3 labeled GSCs (****P ≤ 0.001, ***P ≤ 0.005, **P ≤ 0.01, n.s., nonsignificant (*P* > 0.05)).

### BrdU labeling

We followed previously published methods to identify GSCs in the S phase^92^. Dissected ovaries were incubated in PBS containing 20 μM BrdU (Sigma-Aldrich) for 1 h at RT, washed, and fixed with 5% paraformaldehyde in PBS for 10 min. Ovaries were denatured for 30 min in 2 N HCl, neutralized in 100 mM borax solution (sodium tetraborate) for 2 min, and immunostained with mouse anti-BrdU (1:50; Abcam) as above. GSCs were identified by positive immunostaining with anti-α-Spec (1:100, DSHB) and Vas (1:2000) and their attachment to the cap cells. A Chi-square test was used to analyze counts of BrdU-labeled GSC. (****P ≤ 0.001, ***P ≤ 0.005, **P ≤ 0.01, n.s., nonsignificant (*P* > 0.05)).

### TUNEL assay

Ovaries were dissected in PBS (pH 7.8) and fixed with 4% paraformaldehyde. Ovaries were rinsed twice and washed with PBSX and PBST two times for 10 min each at room temperature, followed by incubation with equilibration buffer (ApopTag® Fluorescein In Situ Apoptosis Detection Kit, Merck Millipore) for 2 min at room temperature. The samples were incubated with the equilibration buffer containing Recombinant Terminal Deoxynucleotidyl Transferase (TdT) for 1 h at 37 °C in the dark. After incubation with stop buffer at RT, the samples were washed three times with PBSX for 10 min. After staining with anti-α-Spectrin (1:100, DSHB) and DAPI, the samples were washed with PBST and mounted. All samples were examined under a fluorescent DIC microscope BX53-34-FL (Olympus). A Chi-square test was used to analyze counts of TUNEL-labeled germarium (****P ≤ 0.001, ***P ≤ 0.005, **P ≤ 0.01, *P ≤ 0.05, n.s., nonsignificant (*P* > 0.05)).

### Measuring developmental rates using labeled clones

We generated mitotic clones in the ovary as previously described^93^. *hs-FLP/*+*; X-15-33/X-15-29* females were produced by standard crosses. To induce FLP expression, female pupae placed in 1.5 mL microcentrifuge tubes were incubated at 37 °C for 1.1 h and placed in testing vials. Heat-shocked pupae were allowed to enclose and were cultured in the vials for one to four days before dissection of the ovary. Clonal cells in germline and somatic tissues were identified by expression of the β-galactosidase reporter as detected by LacZ antibody staining as described above. Both β-galactosidase-positive germline and follicle cells were counted according to the oogenesis stages. Cell division rates of follicle cells were calculated based on the clonal size of follicle epithelial cells at stage 10^20,46^.

### Ex vivo ovary culture

Females cultured in the M+ or M− condition were dissected in Schneider’s insect medium (Gibco). Approximately 8–10 ovaries were transferred to a microcentrifuge tube containing 100–150 μL of Schneider’s medium supplemented with 15% fetal calf serum (MP Biomedicals) and 0.6% penicillin-streptomycin (Gibco) with the different concentrations of synthetic 20E (Tokyo Chemical Industry Co.) or JHIII (Cayman Chemical Company). The ovaries were incubated at RT for 16 h, and then samples were immunostained with anti-pMad and anti-α-Spec to count GSC numbers. A Chi-square test was used to analyze counts of pMad-positive cells for GSC. (****P ≤ 0.001, *P ≤ 0.05, n.s., nonsignificant (*P* > 0.05)).

### Measurement of 20E levels by EIA

Female ovaries maintained under M+ or M- condition were dissected in PBS, homogenized in 50 μL of methanol, and centrifuged at 20,000 × *g* for 1 min. The supernatants were transferred to new tubes and dried with a centrifugal vacuum evaporator. Then, samples were resuspended in 50 μL of EIA buffer and applied to the 96-well incubating plates, following the manufacturer’s protocol (SPI bio) and a previous study^24^. In detail, absorbance was measured at 415 nm using a microplate reader SH-9000Lab (Corona Electric Co., Ltd.) after washing the well with wash buffer and developing with Ellman’s reagent for 100 min. 20E levels in the samples were calculated by the standard curve generated from eight different concentrations of 20E.

### Quantification of receptor activation

*hs-GAL4-Gce; UAS-nlacZ* and *hs-GAL4-Met; UAS-nlacZ* female flies were incubated in a 37 °C block incubator for 1 h after culturing for 3 days in the M+or M- vials and allowed to recover at 25 °C overnight. Animals were dissected and stained with SPiDER-βGal for visualizing and quantifying the fluorescent intensity derived from LacZ. The enzymatic activity of LacZ converts SPiDER-βgal into an intermediate which covalently binds to the surrounding proteins through nucleophilic interaction and produces a fluorescent signal. The fluorescence intensity by SPiDER-βGal is normalized to that of DAPI signal. For statistical analysis, a Wilcoxon rank sum test is used. (***P ≤ 0.005, *P ≤ 0.05, n.s, nonsignificant (*P* > 0.05)).

### Hemolymph extraction

Hemolymph extraction was performed as previously described^82^. For each replicate, hemolymph was collected from fifty virgin females that had been placed in M+ or M− conditions (for 1 or 3 days). The extract was evaporated under N_2_ and stored at −20 °C until all replicates were prepared. Samples were reconstituted in 20 μL of hexane and immediately analyzed by direct analysis in real time mass spectrometry (DART MS).

### Mass spectrometry and data analysis

Mass spectra were acquired with an atmospheric pressure ionization time-of-flight mass spectrometer (AccuTOF-DART 4G, JEOL USA, Inc., Peabody, MA) equipped with a DART SVP ion source (IonSense LLC, Saugus, MA) interface, placed 1 cm away from the sampling orifice. The instrument resolving power is 10,000 (FWHM definition) at *m/z* 500. Voltage settings and acquisition parameters for positive ion mode are as previously described^94^. Calibration for exact mass measurements was performed by acquiring a mass spectrum of polyethylene glycol (average molecular weight 600). Metabolite measurements from hemolymph extract was performed as previously described^82^. The averaged signal intensity of each technical replicate was normalized to intensity of the internal standard (to account for sample loss and sample placement variation) and to the total weight of the hemolymph (to account for variation in hemolymph collecting procedure). The analysis of JH by DART produces two characteristic at [M + H]^+^ 267.20 (intact molecule) and at [M-H_2_O + H]^+^ 249.18 (loss of water), consistent with previous studies of synthetic and natural JH^82,95^. The abundance of the [M-H_2_O + H]^+^ signal was used for all measurements because the parent ion at *m*/*z* 267.20 could not be consistently resolved due to interference from other signals. A Wilcoxon rank sum test was used to analyze relative levels of JH; n.s., nonsignificant (*P* > 0.05).

### Statistics and reproducibility

All experiments were performed independently at least twice and we obtained the robust results similarly. Statistical analysis using a Chi-square test or a Wilcoxon rank sum test for each experiment is described in the figure legends and method sections, respectively. R (version 3.6.3) was used for the statistical analyses with values of *P*  <  0.05 considered as significant. Statistical analysis with no significant differences (*P* > 0.05) is noted “n.s.”. The sources for datasets are provided.

### Reporting summary

Further information on research design is available in the Nature Portfolio Reporting Summary linked to this article.

### Supplementary information

Supplementary Figures

Description of Additional Supplementary Files

Supplementary Data 1

Supplementary Data 2

Reporting Summary

## Data Availability

16S amplicon data sets have been deposited to the DNA Data Bank of Japan (DDBJ). BioProject Accession: PRJDB16024 (PSUB020550). Supplementary Data 1 and Supplementary Data 2 contain individual values underlying the graphs and charts presented in the Figures and Supplementary Figures. All fly strains and materials generated for this study are available upon request.
