# Supplementary material for: Microbes control Drosophila germline stem cell increase and egg maturation through hormonal pathways
Source: Commun Biol. 2023 Dec 20;6:1287. doi: 10.1038/s42003-023-05660-x (PMC10733356; doi:10.1038/s42003-023-05660-x)
Supplement: Supplementary file 1 — Supplementary Figures [file 42003_2023_5660_MOESM1_ESM.pdf]

Supplementary Figure 1.

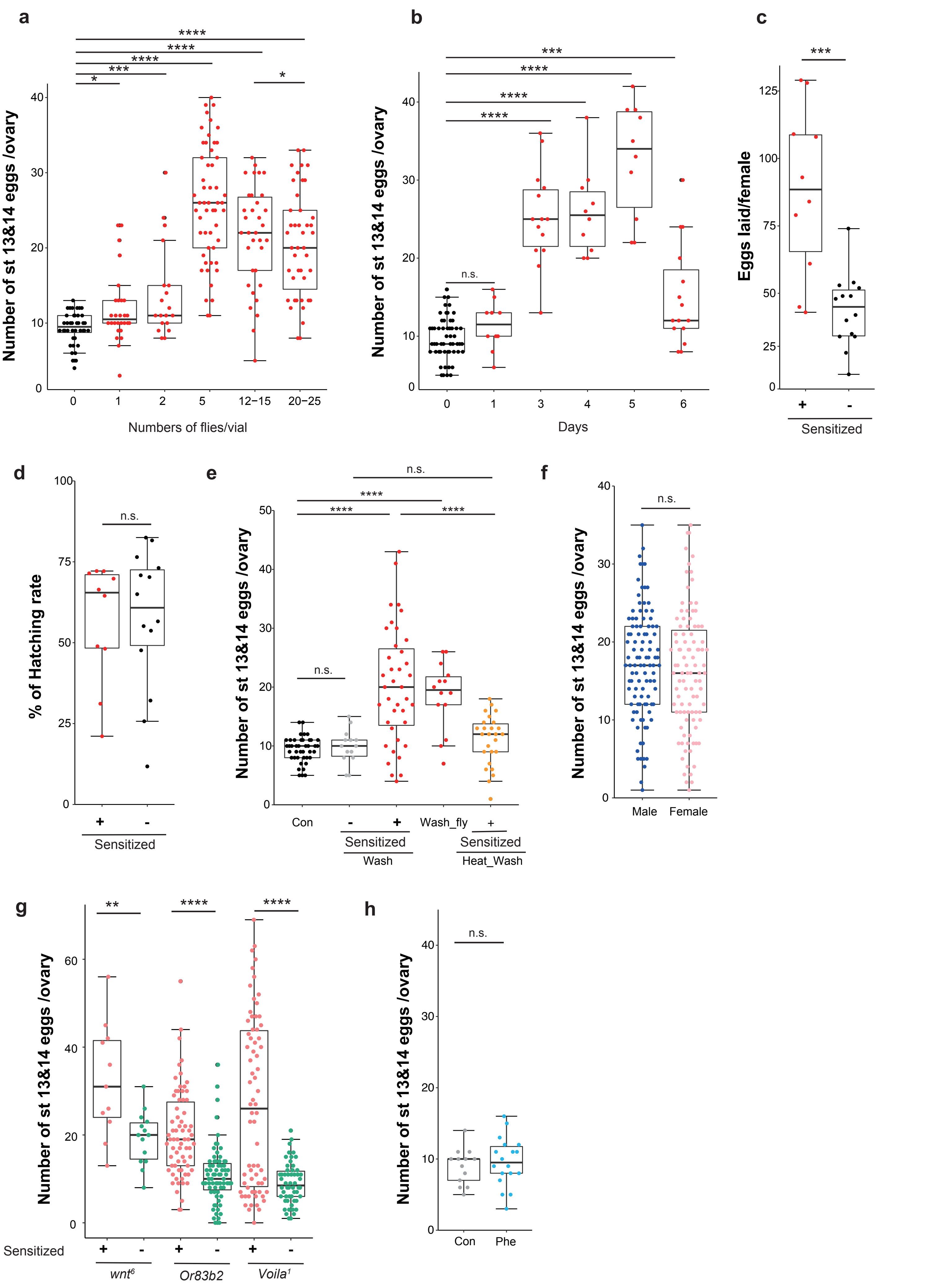

Supplementary Figure 1. Environmental factors enhance mature egg production

a The number of stage 13/14 eggs per ovary from females cultured for 3 days in vials sensitized with varying numbers of males (0 - 25). **b** The number of stage 13/14 eggs per ovary from females cultured in sensitized vials from 0 to 6 days. **c** The number of eggs laid by females cultured in male-sensitized (+) or unsensitized (-) vials. **d** The hatching rate of eggs from females cultured in male-sensitized (+) or unsensitized (-) vials. Females were mated with wildtype males for egg laying and hatching. **e** The number of stage 13/14 eggs per ovary from females cultured for 3 days in vials treated with one of the following conditions: unsensitized (Con), swabbed exudate collected from male-sensitized vials (Wash, sensitized +), swabbed exudate collected from unsensitized vials (Wash, sensitized -), wash solution of the entire fly body (Wash_fly) or heat-inactivated swabbed exudate collected from sensitized vials (Heat_Wash, sensitized +). **f** The number of stage 13/14 eggs per ovary from females cultured in sensitized vials with males or females. **g** The number of stage 13/14 eggs per ovary of chemosensory mutant females (wnt^{6}, *or83b2* or $voila^{1}$) cultured in male-sensitized (+) or unsensitized (-) vials. **h** The number of stage 13/14 eggs per ovary from females cultured in vials sensitized with male cuticular lipid and pheromone extracts (Phe) or solvent control (Con). For statistical analysis, a Wilcoxon rank sum test is used. ****$P \leq 0.001$, ***$P \leq 0.005$, **$P \leq 0.01$, *$P \leq 0.05$, n.s., nonsignificant ($P > 0.05$). Data are represented as mean ± standard deviation.

Supplementary Figure 2.

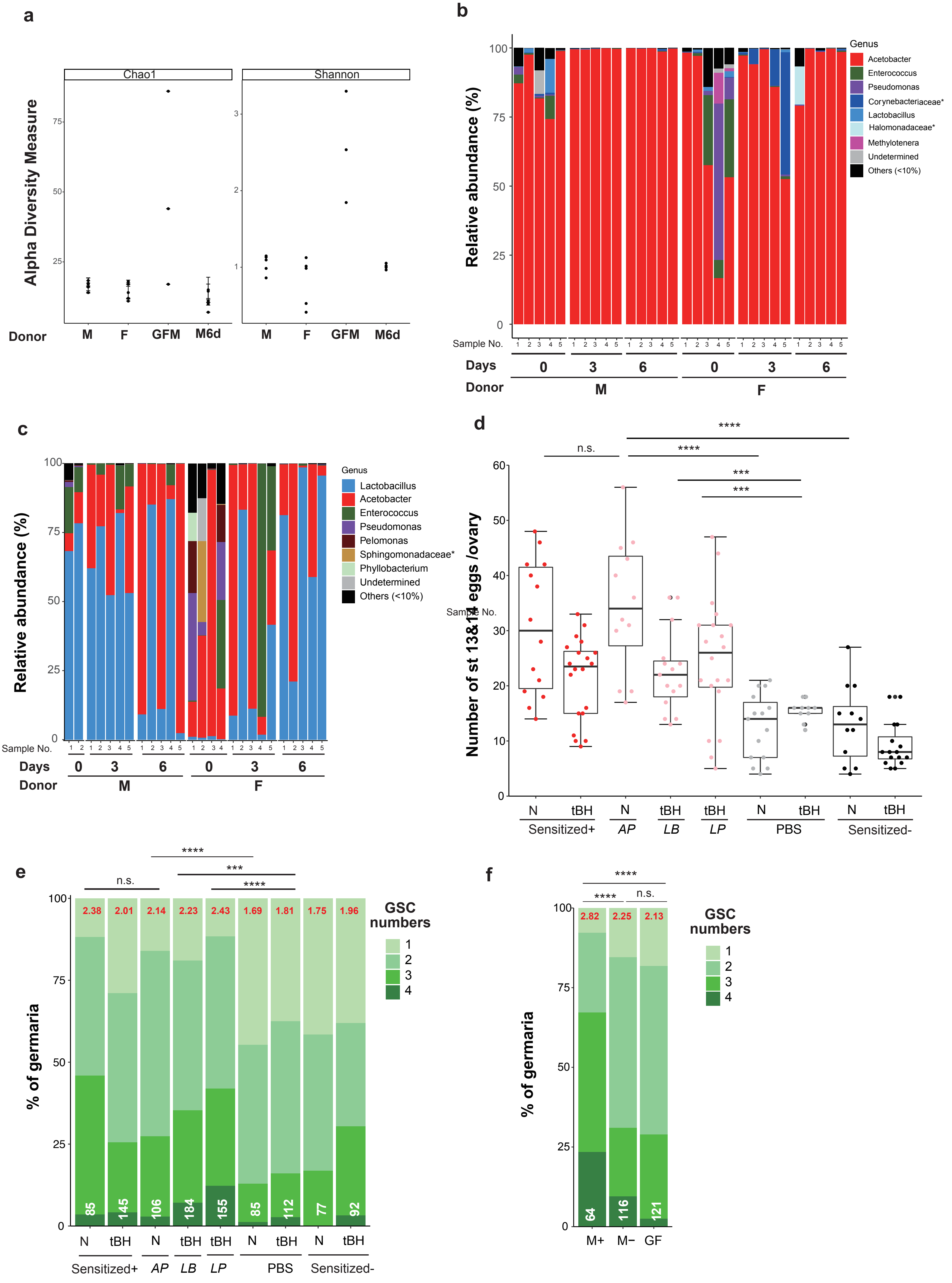

Supplementary Figure 2. Profiles of bacteria deposited on *Drosophila* and the effects on oogenesis

a Alpha diversity plots (Chao1 and Shannon) of microbe composition of flies cultured for 3 or 6 days (M6d) in vials sensitized with one of the following conditions: males (M), females (F) or GF flies (GF). **b** Microbial profiles of male [M] or female [F] flies cultured for 0, 3, or 6 days in vials. **c** Microbiome profiles of laboratory-reared males or females cultured for 0, 3, or 6 days in vials at the Univ. of Hawai'i. Only genera found above 10% relative abundance were used for analysis; unclassified species were denoted by * in panels **b** and **c. d** The number of stage 13/14 eggs per ovary from females cultured for 3 days in vials treated with one of the following conditions: cultured *A. pomorum (AP)*, *L. platinum (LP), L. brevis (LB),* PBS, sensitized+ or sensitized-. Either normal low yeast food (N) or *tBH* containing normal low yeast food (*tBH*) were used. **e** Frequency of germaria containing 1-4 GSCs in females cultured in the conditions used in **d**. GSCs were visualized by immunostaining with pMad antibody for quantification. **f** Frequency of germaria containing 1- 4 GSCs in females cultured in M+, M- or GF flies. The average number of GSCs per germarium and number of germaria examined are shown on the top (red) and the bottom (white), respectively, for **e** and **f**. For statistical analysis, a Wilcoxon test is used for **d** and a Chi-square analysis is used for **e** and **f**. ****$P \leq 0.001$, ***$P \leq 0.005$, n.s., nonsignificant ($P > 0.05$). Data are represented as mean ± standard deviation for **d**.

Supplementary Figure 3.

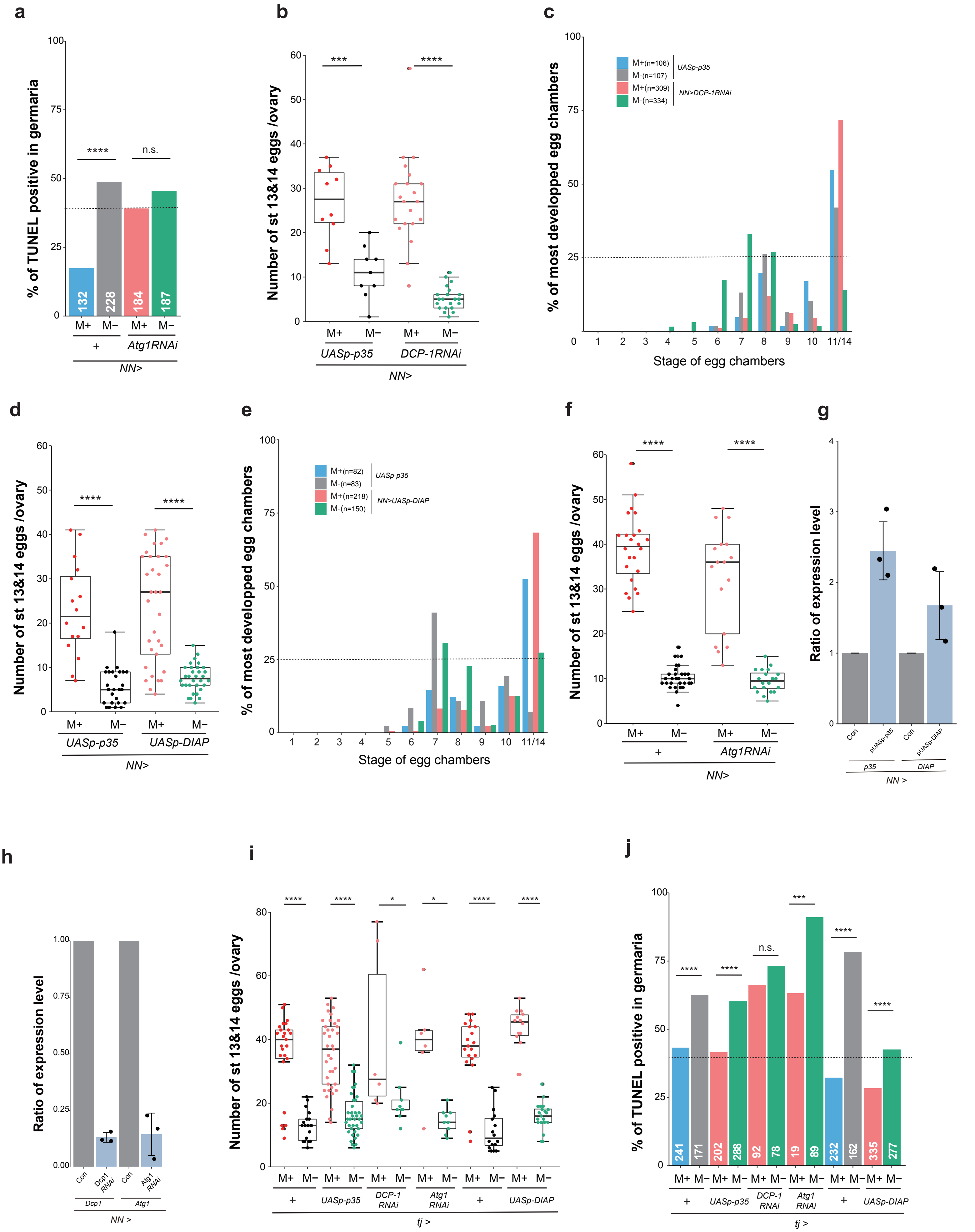

Supplementary Figure 3. Regulation of apoptosis in microbe-induced oogenesis

a The percentage of TUNEL-positive germaria in the indicated females (*NN-Gal4* or *NN-Gal4>Atg1*^{RNAi}) cultured in M+ or M- conditions. **b, d, f** The number of stage 13/14 eggs per ovary from apoptosis- or autophagy-suppressed females cultured in M+ or M- conditions. **b**: *UASp-p35, DCP-1*^{RNAi} ; **d**: *UASp-DIAP* ; or **f**: *Atg*^{RNAi} was driven by *NN-Gal4*. **c, e** The profiles of the most developed egg chambers in the ovarioles of females shown in **b** and **d**. **g, h** Validation by RT-qPCR following over-expression (**g**: *UASp-p35* or *UASp-DIAP*) or knock down (**h** : *DCP-1*^{RNAi} or *Atg1*^{RNAi}) using *NN-Gal4* in ovaries. The mRNA levels of each genotype are quantified in triplicate and normalized with *rp49*. **i** The number of stage 13/14 eggs per ovary from females cultured in M+ or M- conditions. Each of the indicated genes (*UASp-p35*, *DCP-1*^{RNAi}, *Atg1*^{RNAi} or *UASp-DIAP*) was expressed in females by the somatic driver, *tj-Gal4*. **j** The percentage of TUNEL-positive germaria from females shown in **i,** cultured in M+ or M- conditions. The number of germaria examined is shown on the bottom (white) (**a** and **j**). Dotted line indicates 40% (**a** and **j**) or 25% (**c** and **e**) of y-axis. For statistical analysis, a Chi-square analysis is used for **a** and **j**, a Wilcoxon rank sum test is used for **b, d, f** and **i**. ****$P \leq 0.001$ and ***$P \leq 0.005$, *$P \leq 0.05$, n.s., nonsignificant ($P > 0.05$). Data are represented as mean ± standard deviation for **b, d, f** and **i**.

Supplementary Figure 4.

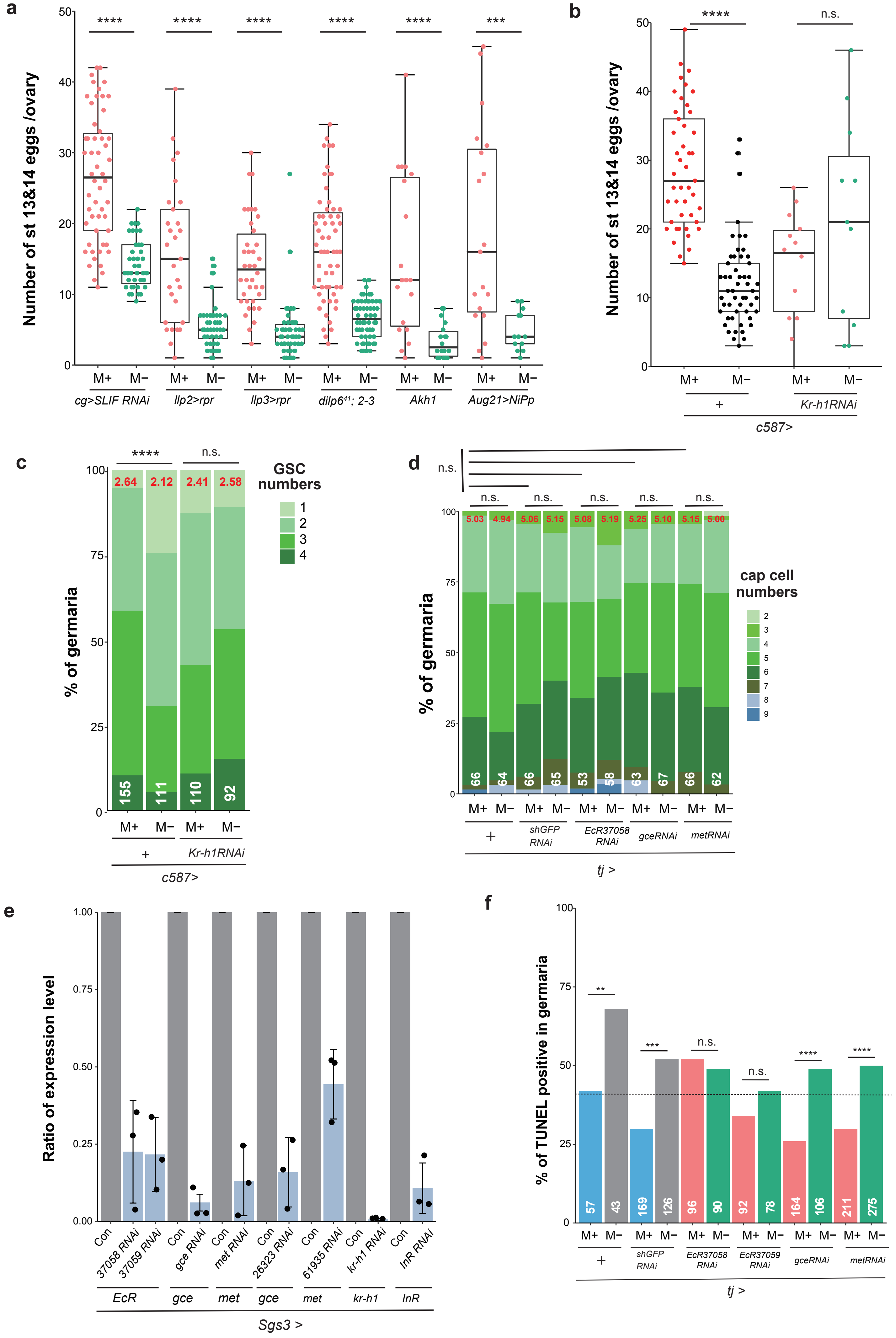

Supplementary Figure 4. Examination of the insulin pathway in microbe-induced oogenesis

a The number of stage 13/14 eggs per ovary from females of the following gentoypes cultured in M+ or M- conditions: knockdown of the *slimfast* amino acid transporter in the fat body (*cg-Gal4>slif*^{RNAi}), elimination *of Drosophila* insulin-like signaling (d*ilp2-Gal4>rpr*, *dilp3-Gal4>rpr,* or *dilp6*^{41}; *2-3*), suppression of adipokinetic hormone (*Akh1*$^{-/-}$) or elimination of JH by expressing *Nuclear Inhibitor of Protein Phosphatase (NiPp)* in corpora allata (*AUG21-Gal4> NiPp*). **b** The number of stage 13/14 eggs per ovary of females (*c587-Gal4,* or *c587-Gal4>Kr-h1*^{RNAi}) cultured in M+ or M- conditions. **c** Frequency of germaria containing 1-4 GSCs of females (*c587-Gal4* or *c587-Gal4>kr-h1* ^{RNAi}*)* cultured in M+ or M- vials. pMad-positive cells next to cap cells were counted as GSCs. The average number of GSCs per germarium and number of germaria examined are shown on the top (red) and the bottom (white), respectively. **d** Frequency of germaria containing 2-9 cap cells of females (*tj-Gal4*, *tj-Gal4 > shGFP*^{RNAi}*, EcR37058*^{RNAi}*, gce*^{RNAi}*, met*^{RNAi}*)* cultured in M+ or M- vials. LaminC-positive cells adjacent to GSCs were counted as cap cells. The average number of cap cells per germarium and number of examined germaria are shown on the top (red) and the bottom (white), respectively. **e** Validation by RT-qPCR of gene knock down in the salivary glands for *EcR37058* ^{RNAi}, *EcR37059*^{RNAi}, *gce*^{RNAi}, *met*^{RNAi}*, gce26323*^{RNAi}*, met61935*^{RNAi}*, kr-h1*^{RNAi} or *InR*^{RNAi} expressed by *Sgs3-Gal4*. The mRNA levels of each genotype are quantified in triplicate and normalized with *rp49*. **f** The percentage of TUNEL-positive germaria from females shown in Fig5EF. The number of examined germaria is shown on the bottom (white). Dotted line indicates 40% of y-axis. For statistical analysis, a Wilcoxon test is used for **a** and **b** and a Chi-square analysis is used for **c, d** and **f**. ****$P \leq 0.001$, ***$P \leq 0.005$, **$P \leq 0.01$, n.s., nonsignificant ($P > 0.05$). Data are represented as mean ± standard deviation for **a, b** and **e**.

Supplementary Figure 5.

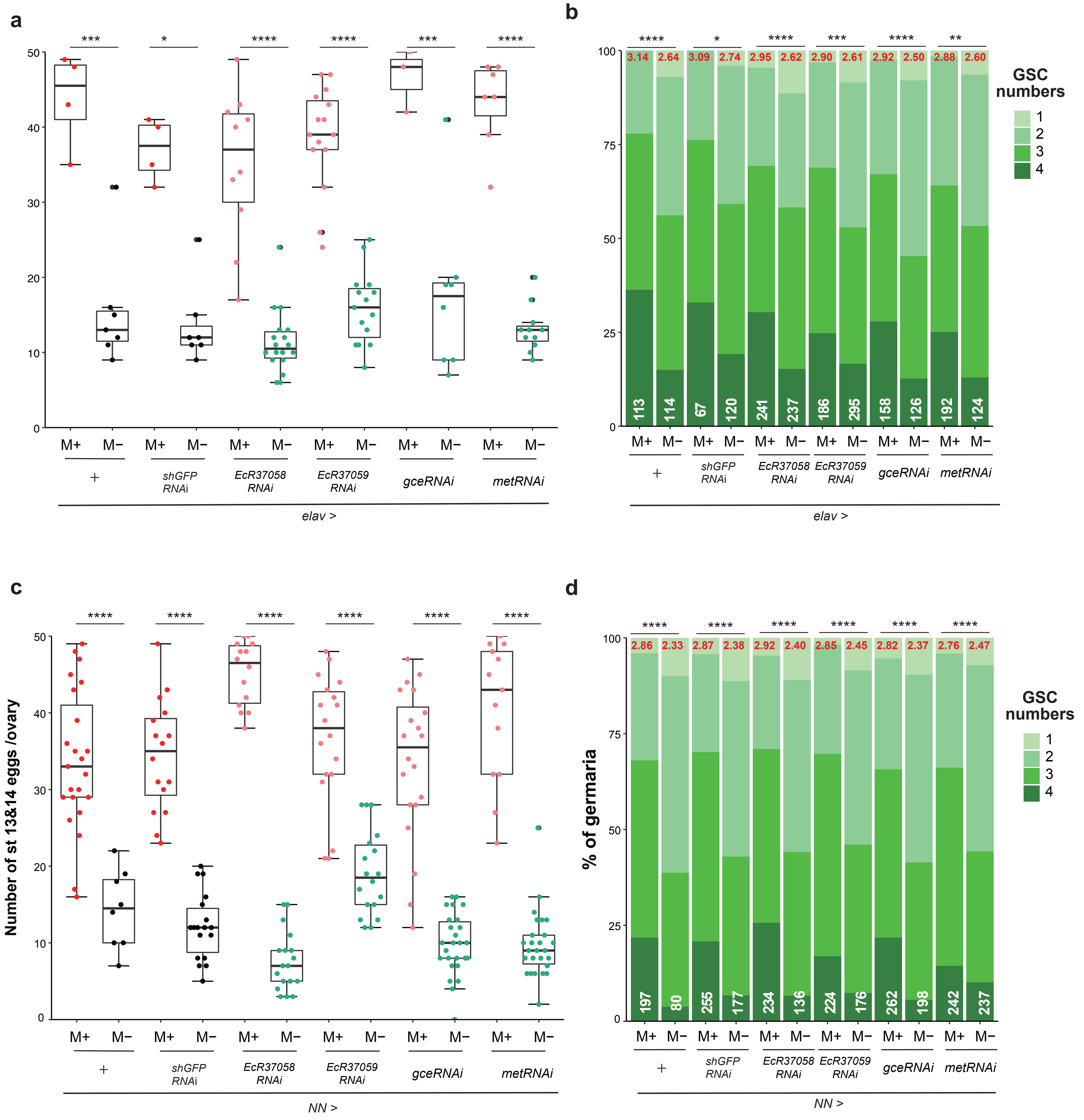

Supplementary Figure 5. Disruption of hormone receptors in neurons or germ cells does not affect microbe-induced oogenesis

a-d Ecdysone or Juvenile hormone pathway-related genes (*EcR37058*^{RNAi}, *EcR37059*^{RNAi}*, gce*^{RNAi}, *met*^{RNAi}) were knocked down with a pan-neuron driver, *elav-Gal4* in **a** and **b**, or by germline specific driver, *NGT40; nos-Gal4-VP16 (NN-gal4)* in **c** and **d**. **a, c** The number of stage 13/14 egg chambers per ovary of indicated females cultured in M+ or M- vials. **b, d** Frequency of germaria containing 1-4 GSCs of indicated females cultured in M+ or M- vials. pMad-positive cells adjacent to cap cells were counted as GSCs. The average number of GSCs per germarium and number of germaria examined are shown on the top (red) and the bottom (white), respectively. For statistical analysis, a Wilcoxon rank sum test is used for panels **a, c** and a Chi-square analysis is used for **b** and **d**. ****$P \leq 0.001$, ***$P \leq 0.005$, **$P \leq 0.01$, *$P \leq 0.05$, n.s., nonsignificant ($P > 0.05$). Data are represented as mean ± standard deviation for **a** and **c**.

Supplementary Figure 6.

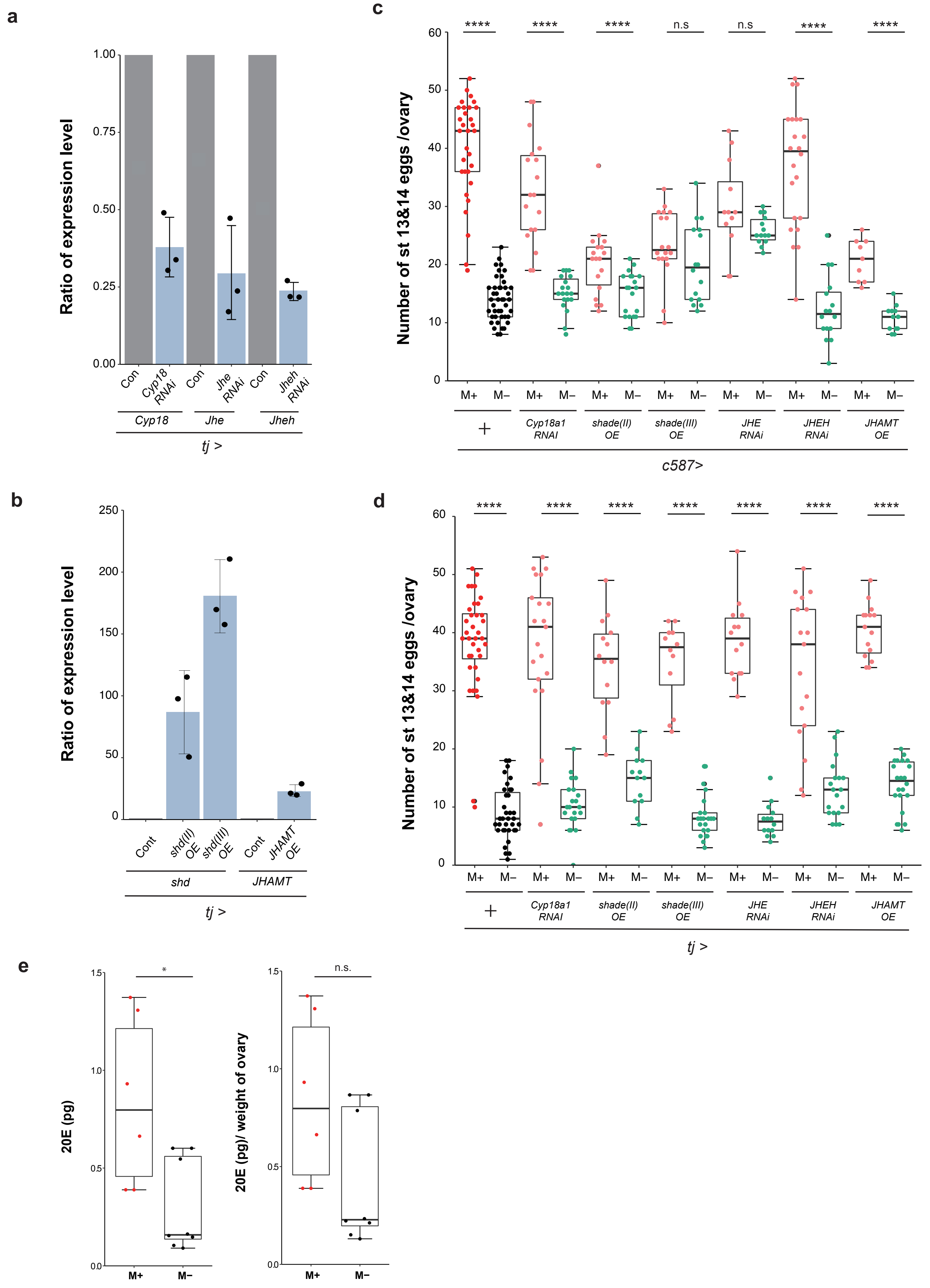

Supplementary Figure 6. Excess levels of 20E and JH do not increase the number of mature eggs.

a, b Validation by RT-qPCR following knock down in ovaries using *tj-Gal4*. The mRNA levels from each genotype were quantified in triplicate and normalized with *rp49*. **c, d** The number of stage 13/14 egg chambers per ovary of females with indicated genotype cultured in either M+ or M- vials; gene expression knock down ($Cyp18^{RNAi}$, jhe^{RNAi} or $jheh^{RNAi}$) and over-expression (*shade(II), shade(III),* or *JHAMT*) were performed using *c587-Gal4* (**c**) or *tj-Gal4* (**d**). **e** 20E levels of ovaries from females maintained under M+ or M- conditions, with (right) or without normalization (left) to the weight of ovaries. For statistical analysis, a Wilcoxon test is used for **c** and **d** ****$P \leq 0.001$, n.s., nonsignificant ($P > 0.05$). Data are represented as mean ± standard deviation.

Supplementary Figure 7.

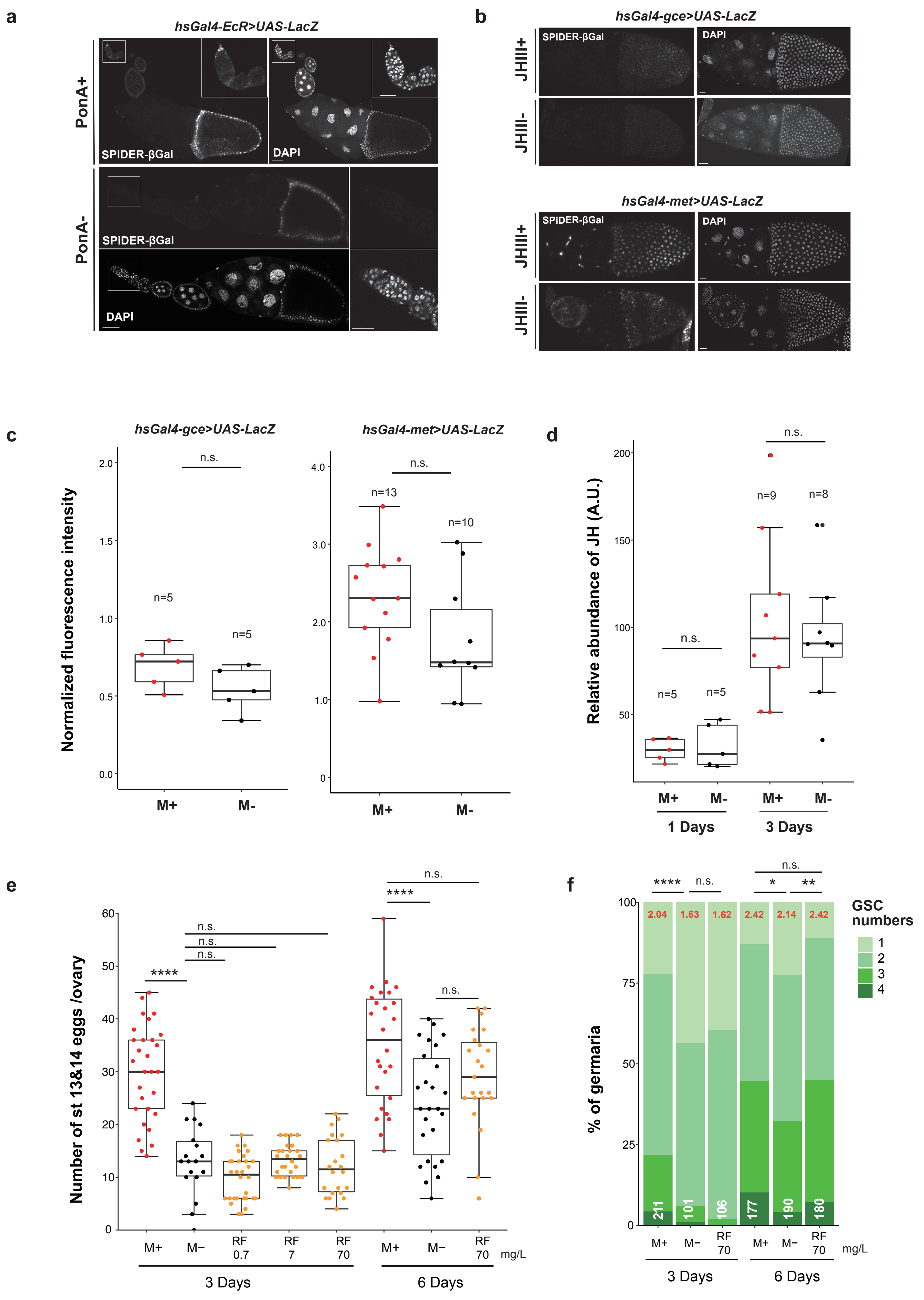

Supplementary Figure 7. Detection of hormone level with reporter system and Riboflavin (RbF)-induced GSC proliferation

a Activation of *hs-Gal4-EcR>UAS-LacZ* in the presence or absence of a synthetic agonist of 20E, Ponasterone A (PonA). The receptor activation was traced with LacZ using SPiDER-β Gal and nuclei were stained by DAPI. **b** Activation of *hs-Gal4-gce>UAS-LacZ* (upper) or *hs-Gal4-met>UAS-LacZ* (lower) in the presence of absence of a synthetic JHIII. The receptor activation was monitored with LacZ activity using SPiDER-βGal and nuclei were stained by DAPI. **c** Females containing *hs-Gal4-gce>UAS-LacZ* (left) *or hs-Gal4-met>UAS-LacZ* (right) were heat shocked and the ovaries dissected for histochemical staining by SPiDER-β Gal and DAPI. The fluorescence intensity of SPiDER-β Gal (514 nm) is normalized to that of DAPI signal (405 nm). **d** The relative abundance of JH from the hemolymph of females cultured in M+ or M- conditions for 1 or 3 days. Each point represents the normalized abundance from 50 females. **e** The number of stage 13/14 eggs per ovary from females cultured in M+ or M- conditions using standard low yeast food or RbF-containing food (final conc. 0.7, 7, 70 mg/L). Females were cultured for 3 or 6 days. **f** Frequency of germaria containing 1-4 GSCs from females cultured in one of the following conditions; M+ or M- using standard low yeast food or RbF-containing food (final conc. 70 mg/L). The average number of GSCs per germarium and number of germaria examined are shown on the top (red) and the bottom (white), respectively. For statistical analysis, a Wilcoxon test is used for **c**, **d** and **e**. a Chi-square analysis was used in F. ****$P \le 0.001$, **$P \le 0.01$, *$P \le 0.05$, n.s., nonsignificant ($P > 0.05$). Data are represented as mean ± standard deviation for **c**, **d** and **e**. Scale bar = 50 μm in **a**, 25 μm in inset of **a**,100 μm in **b**.

Supplemantary Table1

Donor

SF1A	CFU/vial			
Number of flies	Mannitol_Av	SD	MRS_Av	SD
1fly	623700	98319	294300	68890
2fly	1541700	75262	1129140	801592
5fly	7387200	414527	3469467	1337701
12fly	1015200	24746	785700	243405
19fly	1242000	68890	1044900	772648

Donor

SF1B	CFU/fly			
Days	Mannitol_Av	SD	MRS_Av	SD
1D	14580	1406	10200	4604
3D	862650	155002	506722	392683
4D	733500	103073	593325	106673
5D	273845	250800	274050	64589
6D	128250	9664	168345	46312

Donor

F1D	CFU/fly			
Days	Mannitol_Av	SD	MRS_Av	SD
Male	624510	175011	529200	240330
Female	627750	92797	490455	379306
GF	0	-	0	-
6D	119340	11057	133920	99552

Acceptor

F1E	CFU/fly			
Days	Mannitol_Av	SD	MRS_Av	SD
Male	349200	97437	360900	43312
Female	235913	36167	231390	98728
GF	50220	7424	23460	17674
6D	52200	4124	32200	14516
Control	25538	9380	33180	5073

Supplementary Table 2

Reagent/resource	Source	Identifier
Antibodies		
Rabbit anti-Smad3 (EP823Y)(1:1000; IF)	Abcam	Cat# ab52903
Mouse anti-alpha-spectrin (1:100; IF)	DSHB	Cat# DSHB 3A9
Guinia pig anti-Vas (1:2000; IF)	In-house	N/A
Rabbit anti-pH3 (1:400 ; IF)	Millipore	Cat# 06-570
Rat anti-BrdU (1:100; IF)	Becton Dickinson	Cat# ab6326
Mouse anti-LaminC (1:200, IF)	DSHB	Cat# LC28.26
Chickin anti-LacZ antibody (1:200; IF)	Abcam	Cat# ab9361
Chemicals		
SPiDER-βGal	DOJINDO	Cat# SG02
DAPI	Nacarai	Cat#19178-91
5-Bromo-2′-deoxyuridine (BrdU)	Sigma-Aldrich	Cat# B5002
tert-Butyl hydroperoxide solution (tBH)	Sigma-Aldrich	Cat# 458139
Riboflavin (RF)	Wako	Cat# 188-00172
Ampicilin Sodium	Wako	Cat# 014-23302
Tetracycline Hydrochroride	nacalai tesque	Cat# 33031-22
Erythromycin	Wako	Cat# 057-07151
Chloramphenicol	Wako	Cat# 032-19451
Lactobacillus MRS Broth	Becton, Dickinson and Company	Cat# 288130
Lactobacillus MRS Agar	Becton, Dickinson and Company	Cat# 288210
Bacto Peptone	Becton, Dickinson and Company	Cat# 211677
Yeast extract	nacalai tesque	Cat# 15837-55
Mannitol	Wako	Cat# 133-00845
Pentadecanoic acid	Sigma-Aldrich	Cat# W433400
Methanol (HPLC grade)	Fisher Scientific	Cat# A452
Hexane (HPLC grade)	Fisher Scientific	Cat# H302
Juvenile Hormone III	Cayman Chemical	Cat# 19646
Ponasterone A (PonA)	Thermo Fisher Scientific	Cat# H10101
Critical commercial assays		
ApopTag® Fluorescein Direct In Situ Apoptosis Detection Kit	Millipore	Cat# S7160
QIAamp DNA Micro Kit	QIAGEN	Cat# 56304
Pathogen Lysis Tubes L	QIAGEN	Cat# 19092
MagAttract PowerSoil DNA EP Kit	QIAGEN	Cat# 27100-4-EP
PowerMag Microbial DNA Isolation Kit	QIAGEN	Cat# 27200
KAPA3G Plant kit	Sigma Aldrich	Cat# KK7251
Just-a-plate 96 PCR Normalization and Purification Kit	Charm Biotech	Cat# JN-120-10
Anaero Pack Pouch	Mitsubishi gas chemical	Cat# A-213
Puritan™ PurFlock™ Ultra Flocked Swabs	Puritan™ 253306U	Cat# 22-025-192
KAPA SYBR Fast qPCR Kit	KAPA BIOSYSTEMS	Cat# KK4605
20-Hydroxyecdysone Enzyme Immunoassay kit	SPIBIO	Cat# A05120
Plasmids		
hs-Gal4gceLBD	This study	N/A
hs-Gal4metLBD	This study	N/A
Fly strains (*D.melanogaster*)		
Canton-S-iso2G	BDSC	BDSC Cat# 9515; BDSC_9515
hs-FLP; X-15-29; EC34/TM6 Tb	Margolis1995; Drummond-Barbosa2001	N/A
y w; X-15-33/CyO	Margolis1995; Drummond-Barbosa2001	N/A
UASp-p35/CyO	Werz2005	N/A
y[1] sc[*] v[1] sev[21]; P{y[+t7.7] v[+t1.8]=TRiP.HMS01779}attP2	BDSC	BDSC Cat# 38315; BDSC_38315
y[1] w[*]; P{w[+mC]=UASp-Diap1.P}9-4	BDSC	BDSC Cat# 63820; BDSC_ 63820
y[1] v[1]; P{y[+t7.7] v[+t1.8]=TRiP.HMS02750}attP40	BDSC	BDSC Cat# 44034; BDSC_ 44034
y[1] sc[*] v[1] sev[21]; P{y[+t7.7] v[+t1.8]=TRiP.HMS01358}attP2/TM3, Sb[1]	BDSC	BDSC Cat# 34369; BDSC_ 34369
w^{1118}; P{GD1428}v37058	VDRC	VDRC ID# 37058
w^{1118}; P{GD1428}v37059	VDRC	VDRC ID# 37059
w^{1118}; P{GD104}v991/TM3	VDRC	VDRC ID# 991
P{KK109764}VIE-260B	VDRC	VDRC ID# 101814
P{KK104562}VIE-260B	VDRC	VDRC ID# 100638
y^1 v^1; P{TRiP.JF02097}attP2	BDSC	BDSC Cat# 26323; BDSC_ 26323
y^1 v^1; P{TRiP.HMJ23518}attP40	BDSC	BDSC Cat# 61935; BDSC_ 61935

P{KK112003}VIE-260B	VDRC	VDRC ID# 107935
y[1] sc[*] v[1] sev[21]; P{y[+t7.7] v[+t1.8]=TRiP.HMC05796}attP2	BDSC	BDSC Cat# 64923; BDSC_ 64923
UAS-shd-4A2 (II)	Petryk 2003	N/A
UAS-shd-68A3 (III)	Petryk 2003	N/A
y[1] v[1]; P{y[+t7.7] v[+t1.8]=TRiP.HMJ21834}attP40	BDSC	BDSC Cat# 57826; BDSC_ 57826
y[1] v[1]; P{y[+t7.7] v[+t1.8]=TRiP.HMC03077}attP2	BDSC	BDSC Cat# 50676; BDSC_ 50676
UAS-DmJHAMT (II)	NIWA 2008	N/A
w*; P{UAS-NiPp1.HA}3/TM6C, cu^{1} Sb^{1}	BDSC	BDSC Cat# 23711; BDSC_ 23711
y[1] sc[*] v[1] sev[21]; P{y[+t7.7] v[+t1.8]=TRiP.HMC05846}attP40	BDSC	BDSC Cat# 64972; BDSC_ 64972
w[1118]; TM3, Sb[1]/TM6B, P{w[+mC]=UAS-rpr.C}3, Tb[1]	BDSC	BDSC Cat# 50791; BDSC_ 50791
Df(1)Ilp6[41], y[1] Ilp6[41] Raf[41] w[1118]; Df(3L)Ilp2-3, TI{w[+mW.hs]=TI}Ilp2-3 TI{w[+mW.hs]=TI}Ilp5[4]/TM3, Sb[1]	BDSC	BDSC Cat# 30892; BDSC_ 30892
Akh1	Zemanová 2016	N/A
w[*]; TI{w[+mW.hs]=TI}Orco[2]	BDSC	BDSC Cat# 23129; BDSC_ 23129
w[1118]; TI{w[+mW.hs]=TI}Wnt6[KO]	BDSC	BDSC Cat# 76311; BDSC_ 76311
Voila1/ Tm3, SbSer	Balakireva 1998	N/A
w; NGT40; nos-Gal4-VP16	Tracey 1999	N/A
P{w[+mC]=GAL4-elav.L}2/CyO	BDSC	BDSC Cat# 8765;RRID:BDSC_8765
P{w[+mW.hs]=GawB}C587, w[*]	BDSC	BDSC Cat# 67747;RRID:BDSC_67747
P{GawB}NP1624	DGRC	DGRC Cart# 10455
w[1118]; P{Sgs3-Gal4.PD}TP1	BDSC	BDSC Cat# 6870; BDSC_6870
w; P{GawB}Aug21/CyO*	BDSC	BDSC Cat# 30137;BDSC_30137
w[1118]; P{w[+mC]=Cg-GAL4.A}2	BDSC	BDSC Cat# 7011;BDSC_7011
w[*]; P{w[+mC]=Ilp2-GAL4.R}2	BDSC	BDSC Cat# 37516; BDSC_37516
w1118; P{Ilp3-GAL4.C}2/CyO	BDSC	BDSC Cat# 52669; BDSC_52660
w[1118]; TM3, Sb[1]/TM6B, P{w[+mC]=UAS-rpr.C}3, Tb[1]	BDSC	BDSC Cat# 50791; BDSC_50791
w[1118]; P{w[+mC]=hs-GAL4-EcR.LBD}SBM	BDSC	BDSC Cat# 23656; BDSC_23656
w[1118]; P{w[+mC]=hs-GAL4-EcR.LBD}SA	BDSC	BDSC Cat# 23657; BDSC_23657
w[1118]; P{w[+mC]=UAS-lacZ.NZ}20b	BDSC	BDSC Cat# 3955; BDSC_3955
w[1118]; P{w[+mC]=UAS-lacZ.NZ}J312	BDSC	BDSC Cat# 3956; BDSC_3956
Microbes strains		
Acetobacter pomorum NBRC 108911	Shin 2001	N/A
Lactobacillus brevis EW	Shin 2001	N/A
Lactobacillus plantarum WJL	Shin 2001	N/A
Software	Source	Identifier
Fiji (version 2.3.0/1.53q)	Schindelin et al. 2012	https://fiji.sc
R (version 3.6.3)	RStudio Team, 2015	https://www.r-project.org/
phyloseq (version 1.22.3)	McMurdie 2013	https://joey711.github.io/phyloseq/
JEOL MassCenter software (version 1.6.10.04)	JEOL USA	
MassMountaineer (version 5.1.13.0)	RBC Software;Diablo Analytical	

Supplementary Table 3

Target genes	Sequence
ecdysoneR	ACTCCAGCCACAGATTCAACCACA
	CATGTATTCGCTGCTCGTACTGAC
gce	CCAGGAGTGACCAACTCACG
	CATATTCGGATAGAAACCAGGCG
met	GCCTCAAGGGAACGGGTATG
	AGCAGTTGCATTAGAGTGTCC
krh1	CACCTCACACACCAAGTCG
	TGCACTGATAGGGATCTGCTC
InR	AAGCGTGGGAAAATTAAGATGGA
	GGCTGTCAACTGCTTCTACTG
cyp18a1	TACAAGATGATCCGCGAGTG
	ACAGTTTGCCAGTGCTGTTG
Jhe	CAAGTTTGGAAAACCCAGGA
	GGTAGGCATCTGGTGCATTT
JHEH	GCGGGAGTTTTACGATTTCA
	CCTGTGACCAGCCATAACCT
shade	CTGCAGGATGCACTAACGAA
	CCTGCATTAAGCGAGTAGCC
jhamt	ATTCTGGACGAATTCGCATC
	GGAGCAGTGGCTTTACGAAG
rp49	GACGCTTCAAGGGACAGTATCTG
	AAACGCGGTTCTGCATGA
atg1	TGGTGCAAAAGTGCAATCAT
	TAGCTCTATCCGCCCGAGTA
DCP1	GACCTGCTCACCCTGCTTAC
	GTGAGGCACGGTATTTGCTT
DIAP1	TGTGACAAAGTGTCCGCTGT
	CCGCCCACATTTTCTTTTTA
p35	GCAAGGAATCGGATCTTCAG
	ACCAAAAACGGTTTGAGTGG
GAL4_FW	GAATTGGGAATTCAATAATACGACTCACTATAG[illegible]
GAL4_RV	GAATTCCGGCGATACAGTCAACTGTCTTTGACC[illegible]
VFW	CGGTAAATATAAAATTTTTAATGTATAAATGTGT[illegible]
VRV	TTGAATTCCCAATTCCCTATTCAGAGTTCTC
Gce_Fw	GTATCGCCGGAATTCCCACAGATCACGGACAC[illegible]
GAL4_GceRV	ATTTTATATTTACCCTAGTCCTGGTCGTCCTCC[illegible]
Met_Fw	GTATCGCCGGAATTCGGCCTCAAGGGAACGG[illegible]
GAL4_Met_RV	ATTTTATATTTACCGTCATCGCAGCGTGCTGGT
