# Supplementary material for: Microbes control Drosophila germline stem cell increase and egg maturation through hormonal pathways
Source: Commun Biol. 2023 Dec 20;6:1287. doi: 10.1038/s42003-023-05660-x (PMC10733356; doi:10.1038/s42003-023-05660-x)
Supplement: Supplementary file 2 — Description of Additional Supplementary Files [file 42003_2023_5660_MOESM2_ESM.pdf]

Description of Additional Supplementary Files

File name: Supplementary Data 1
Description: The source data behind the graphs in the main figures.

File name: Supplementary Data 2
Description: The source data behind the graphs in the supplementary figures.
