# Supplementary material for: Microbes control Drosophila germline stem cell increase and egg maturation through hormonal pathways
Source: Commun Biol. 2023 Dec 20;6:1287. doi: 10.1038/s42003-023-05660-x (PMC10733356; doi:10.1038/s42003-023-05660-x)
Supplement: Supplementary file 5 — Reporting Summary [file 42003_2023_5660_MOESM5_ESM.pdf]

nature portfolio

Corresponding author(s): Ritsuko SUYAMA, Joanne Y Yew, Toshie KAI

Last updated by author(s): Jun 8, 2023

Reporting Summary

Nature Portfolio wishes to improve the reproducibility of the work that we publish. This form provides structure for consistency and transparency in reporting. For further information on Nature Portfolio policies, see our Editorial Policies and the Editorial Policy Checklist.

Statistics

For all statistical analyses, confirm that the following items are present in the figure legend, table legend, main text, or Methods section.

n/a	Confirmed	
☐	☒	The exact sample size (*n*) for each experimental group/condition, given as a discrete number and unit of measurement
☐	☒	A statement on whether measurements were taken from distinct samples or whether the same sample was measured repeatedly
☐	☒	The statistical test(s) used AND whether they are one- or two-sided<br>*Only common tests should be described solely by name; describe more complex techniques in the Methods section.*
☐	☒	A description of all covariates tested
☐	☒	A description of any assumptions or corrections, such as tests of normality and adjustment for multiple comparisons
☐	☒	A full description of the statistical parameters including central tendency (e.g. means) or other basic estimates (e.g. regression coefficient) AND variation (e.g. standard deviation) or associated estimates of uncertainty (e.g. confidence intervals)
☐	☒	For null hypothesis testing, the test statistic (e.g. *F*, *t*, *r*) with confidence intervals, effect sizes, degrees of freedom and *P* value noted<br>*Give P values as exact values whenever suitable.*
☒	☐	For Bayesian analysis, information on the choice of priors and Markov chain Monte Carlo settings
☒	☐	For hierarchical and complex designs, identification of the appropriate level for tests and full reporting of outcomes
☒	☐	Estimates of effect sizes (e.g. Cohen's *d*, Pearson's *r*), indicating how they were calculated

Our web collection on statistics for biologists contains articles on many of the points above.

Software and code

Policy information about availability of computer code

Data collection	Identification of microbiome was done with MetaFlow|mics (https://dl.acm.org/doi/10.1145/3311790.3396664)
Data analysis	16S amplicon analysis was done mainly with MetaFlow|mics (https://github.com/hawaiidatascience/metaflowmics) including DADA2(https://github.com/benjjneb/dada2) , LULU ((https://github.com/tobiasgf/lulu) and MOTHUR (http://www.mothur.org)<br>16S amplicon analysis for visualizing comparing microbiome profiles was done with phyloseq (https://joey711.github.io/phyloseq/) and xvtyzn/nyankomicro (https://rdrr.io/github/xvtyzn/nyankomicro/)<br>Quantitative analysis of fluorescent signal was done with Image J, Fiji (version 2.3.0/1.53q) (https://fiji.sc)<br>Mass spectrometry analysis was done with JEOL MassCenter software (version 1.6.10.04, JEOL USA) and MassMountaineer (version 5.1.13.0, RBC Software Diablo Analytical)

For manuscripts utilizing custom algorithms or software that are central to the research but not yet described in published literature, software must be made available to editors and reviewers. We strongly encourage code deposition in a community repository (e.g. GitHub). See the Nature Portfolio guidelines for submitting code & software for further information.

Data

Policy information about availability of data

All manuscripts must include a data availability statement. This statement should provide the following information, where applicable:

- Accession codes, unique identifiers, or web links for publicly available datasets
- A description of any restrictions on data availability
- For clinical datasets or third party data, please ensure that the statement adheres to our policy

16S amplicon data sets have been deposited to the DNA Data Bank of Japan (DDBJ). BioProject Accession: PRJDB16024 (PSUB020550).

Research involving human participants, their data, or biological material

Policy information about studies with human participants or human data. See also policy information about sex, gender (identity/presentation), and sexual orientation and race, ethnicity and racism.

Reporting on sex and gender	This research involves no human research participants.
Reporting on race, ethnicity, or other socially relevant groupings	This research involves no human research participants.
Population characteristics	This research involves no human research participants.
Recruitment	This research involves no human research participants.
Ethics oversight	This research involves no human research participants.

Note that full information on the approval of the study protocol must also be provided in the manuscript.

Field-specific reporting

Please select the one below that is the best fit for your research. If you are not sure, read the appropriate sections before making your selection.

☒ Life sciences ☐ Behavioural & social sciences ☐ Ecological, evolutionary & environmental sciences

For a reference copy of the document with all sections, see nature.com/documents/nr-reporting-summary-flat.pdf

Life sciences study design

All studies must disclose on these points even when the disclosure is negative.

Sample size	At least 10 individual biological sample sizes were proved to be sufficient for the evaluation of the germ line development phenotype by statistics analysis. The sample sizes was defined by the pre-examined in the same experimental system. Other experiments including quantification of mRNA were performed by three individual biological samples.
Data exclusions	No data were excluded from the analysis. The results were quantified and compared between conditions by statistical analysis.
Replication	Experiments were performed with at least 2 biological replicates. The reproducibility was also confirmed.
Randomization	Flies were collected based on age and sex but otherwise randomly selected for each condition and genotype used in the experiments.
Blinding	Blinding is not relevant to the evaluation of analysis in sub-cellular localization, morphology and functional analysis due to the definitive genotype of Drosophila.

Reporting for specific materials, systems and methods

We require information from authors about some types of materials, experimental systems and methods used in many studies. Here, indicate whether each material, system or method listed is relevant to your study. If you are not sure if a list item applies to your research, read the appropriate section before selecting a response.

Materials & experimental systems

n/a	Involved in the study	
☐	☒	Antibodies
☒	☐	Eukaryotic cell lines
☒	☐	Palaeontology and archaeology
☐	☒	Animals and other organisms
☒	☐	Clinical data
☒	☐	Dual use research of concern
☒	☐	Plants

Methods

n/a	Involved in the study	
☒	☐	ChIP-seq
☒	☐	Flow cytometry
☒	☐	MRI-based neuroimaging

Antibodies

Antibodies used

Anti-Smad3 rabbit monoclonal antibody(EP823Y) Abcam, Cat# ab52903
Anti-Vas guinea pig polyclonal antibody, DOI:10.1016/j.cub.2010.02.046
Anti-alpha-spectrin mouse monoclonal antibody,DSHB,Cat# DSHB 3A9
Anti-pH3 rabbit polyclonal antibody, Millipore, Cat# 06-570
Anti-BrdU rat monoclonal antibody, Becton Dickinson, Cat# ab6326
Anti-LacZ chicken polyclonal antibody, Abcam, Cat# ab9361

Validation

Anti-Smad3 rabbit monoclonal antibody(EP823Y) Abcam Cat# ab52903 is validated by company and described in https://www.abcam.co.jp/products/primary-antibodies/smad3-phospho-s423--s425-antibody-ep823y-ab52903.html
Anti-Vas guinea pig polyclonal antibody, validated in DOI:10.1016/j.cub.2010.02.046.
Anti-alpha-spectrin mouse monoclonal antibody,DSHB,Cat# DSHB 3A9 is validated by company and described in https://dshb.biology.uiowa.edu/3A9-323-or-M10-2-
Anti-pH3 rabbit polyclonal antibody, Millipore, Cat# 06-570 is validated by company and described in https://www.sigmaaldrich.com/JP/ja/product/mm/06570
Anti-BrdU rat monoclonal antibody, Becton Dickinson Cat# ab6326 is validated by company and described in https://www.abcam.co.jp/products/primary-antibodies/brdu-antibody-bu175-icr1-proliferation-marker-ab6326.html
Anti-LacZ chicken polyclonal antibody, Abcam, Cat# ab9361 is validated by company and described in https://www.abcam.co.jp/products/primary-antibodies/beta-galactosidase-antibody-ab9361.html

Animals and other research organisms

Policy information about studies involving animals; ARRIVE guidelines recommended for reporting animal research, and Sex and Gender in Research

Laboratory animals

This research involves Drosophila melanogaster. Drosophila used in the study were obtained from the Bloomington Drosophila Stock Center at Indiana University, Bloomington, USA or Drosophila Genetic Resource Center at Kyoto Institute of Technology, Japan.

Wild animals

No wild animals were involved in this study.

Reporting on sex

We clearly show in the abstract that we investigated about female reproduction, germline stem cells, and egg development in female flies. Gender is indicated for all experiments and results.

Field-collected samples

No field-collected samples were involved in this study.

Ethics oversight

No ethics approval or guidance was required for the invertebrate animal.

Note that full information on the approval of the study protocol must also be provided in the manuscript.
